# Physical cues in biomaterials modulate macrophage polarization for bone regeneration: a review

**DOI:** 10.3389/fbioe.2025.1640560

**Published:** 2025-07-23

**Authors:** Tianxiao Yang, Zhurun Fang, Jin Zhang, Shengnai Zheng

**Affiliations:** Department of Orthopaedic Surgery, Institute of Digital Medicine, Nanjing First Hospital, Nanjing Medical University, Nanjing, China

**Keywords:** biomaterials, bone regeneration, macrophage polarization, tissue engineering, physical cues, bone scaffolds

## Abstract

Bone regeneration is a complex process governed by inflammation, angiogenesis, and tissue remodeling. Macrophages play central roles by dynamically shifting between pro-inflammatory (M1) and anti-inflammatory (M2) phenotypes. While biochemical signals have been widely studied, emerging evidence highlights the immunomodulatory potential of physical cues from biomaterials. This review summarizes macrophage functions across bone healing phases and critically examines how physical cues—such as stiffness, topography, pore architecture, hydrophilicity, electromagnetic stimuli, and metal composition—modulate macrophage polarization. We discuss underlying mechanosensing mechanisms, phenotype plasticity, and the dynamic interplay between materials and immune cells. Finally, we highlight current limitations and propose future directions to guide the design of next-generation osteo-immunomodulatory biomaterials.

## 1 Introduction

Bone is a dynamic and self-renewing tissue that continuously remodels in response to physiological and mechanical stimuli across the lifespan. Despite its regenerative capacity, bone remains susceptible to trauma, degenerative diseases, and age-related disorders that impair structural integrity. These conditions significantly affect patient quality of life and rank among the leading causes of global disability-adjusted life years (GBD, 2019 Diseases and Injuries Collaborators et al., 2020).

Beyond their classical role in host defense, macrophages have attracted growing interest in their multifaceted involvement in bone regeneration. These versatile immune cells orchestrate the complex crosstalk among inflammation, matrix remodeling, and immune signaling that ultimately determines regenerative outcomes (Schlundt et al., 2018). A deeper understanding of macrophage behavior during bone repair is therefore essential for the rational design of immunomodulatory biomaterials in bone tissue engineering.

Macrophages reside in nearly all tissues and act as first-line responders to tissue damage and environmental signals (Ovchinnikov, 2008). Upon injury, they are rapidly recruited to damaged sites via leukocyte extravasation, where they perform key functions, including phagocytosis (Aderem and Underhill, 1999), cytokine release, and antigen presentation (Mann and Li, 2014). Functionally, macrophages can polarize toward classically pro-inflammatory (M1) or anti-inflammatory (M2) phenotypes, depending on local microenvironmental cues (Yunna et al., 2020). These phenotypes exhibit opposing functions—M1 cells are beneficial to pathogen clearance, while M2 cells facilitate bone tissue repair and regeneration.

**FIGURE 1:**
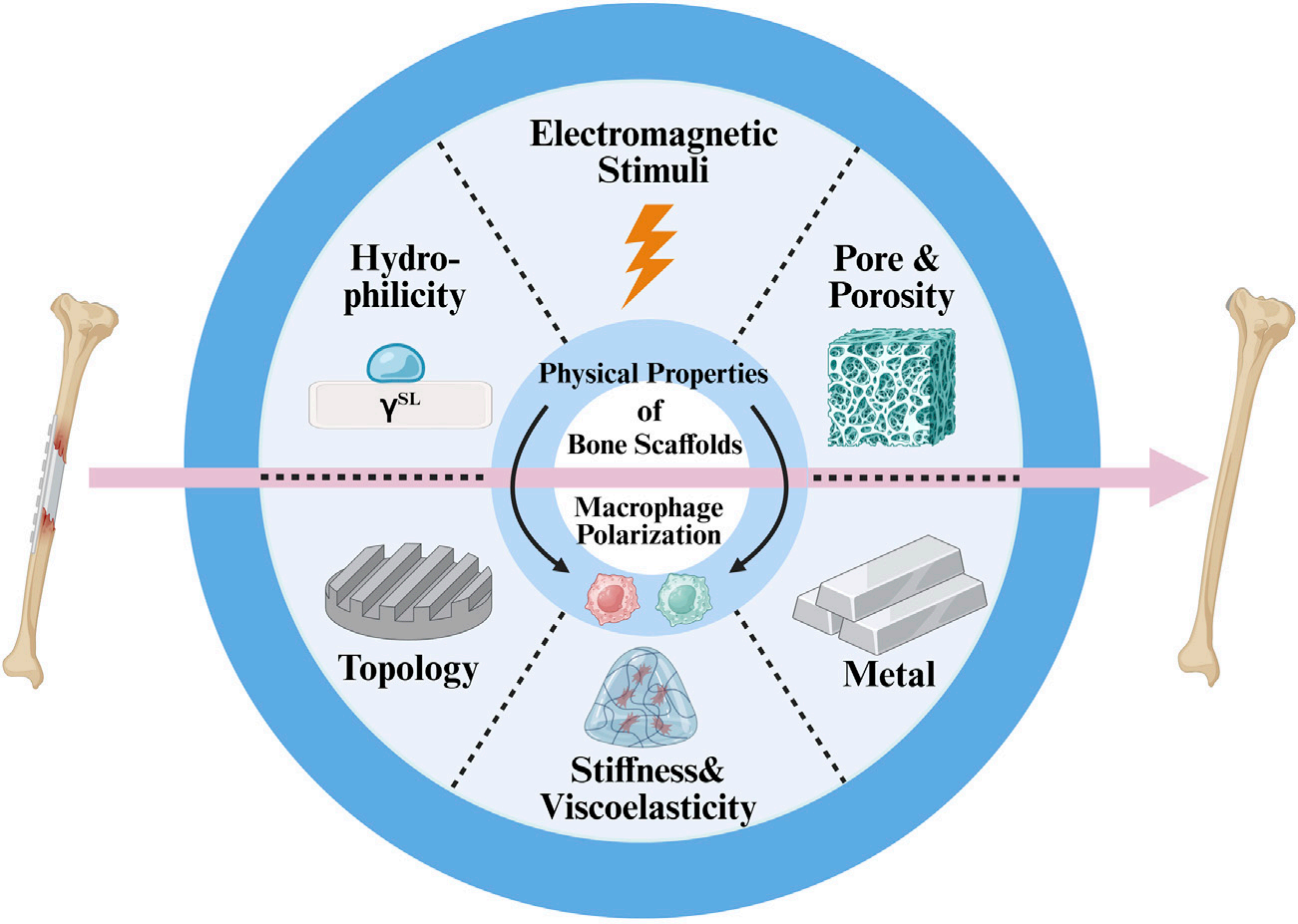
Schematic diagram of key physical cues of bone scaffolds that modulate macrophage polarization and promote bone regeneration.

Recent advances in bone tissue engineering have focused on scaffold-based approaches to enhance osteogenesis. While most strategies emphasize biochemical cues, emerging evidence highlights that the physical cues from biomaterials can also profoundly influence immune cell behavior (Figure 1).

While this review primarily focuses on macrophages, we acknowledge that other immune cells, such as neutrophils and T cells, also play crucial roles in the orchestration of bone regeneration. Neutrophils are among the earliest responders at the injury site, facilitating debris clearance and initiating inflammatory cascades (Bouchery and Harris, 2019; de Oliveira et al., 2016). T cells contribute to immune modulation and osteogenic regulation through cytokine production and interaction with osteoprogenitor cells (Hu et al., 2024; Zhu et al., 2024). However, macrophages remain the most extensively studied immune cells in the context of biomaterial-mediated bone regeneration, with clearly defined phenotypes and mechanosensitive properties. Therefore, this review centers on the immunomodulatory roles of macrophages in response to physical cues, while calling for future work to explore the multicellular immune landscape.

The uniqueness of this review lies in its comprehensive and mechanistically focused synthesis of how the physical properties of biomaterials modulate the behavior of macrophages and thereby influence bone regeneration progress. Unlike prior reviews, we emphasize an emerging paradigm in which scaffold microarchitecture, stiffness, topography, and dynamic mechanical stimuli act not merely as passive supports but as active immuno-instructive elements to shape the bone regenerative outcomes.

## 2 The role of macrophage in bone regeneration

### 2.1 Overview of macrophage polarization and functions

Macrophages are a diverse population of mononucleated phagocytic cells, ubiquitously present across nearly all tissues and extensively involved in a broad spectrum of physiological and pathological processes. These include tissue development, homeostasis (Wynn et al., 2013), immune defense, and regeneration (Locati et al., 2020).

Tissue-resident macrophages, a kind of specialized macrophage subset that permanently resides within specific tissues (Nobs and Kopf, 2021), have distinct developmental origins. For example, bone-resident macrophages, known as osteomacs, originate from yolk-sac erythromyeloid progenitors (Schulz et al., 2012). F4/80 is broadly expressed in tissue-resident macrophages in mice and is therefore commonly used in combination with other markers, such as CD169, to localize and characterize osteomacs (Dos Anjos Cassado, 2017). Notably, F4/80 expression varies significantly among mononuclear phagocyte populations and is either low or absent in osteoclasts, alveolar macrophages, classical dendritic cells, and macrophages located in T cell zones and marginal zones (Gordon et al., 2011).

**FIGURE 2:**
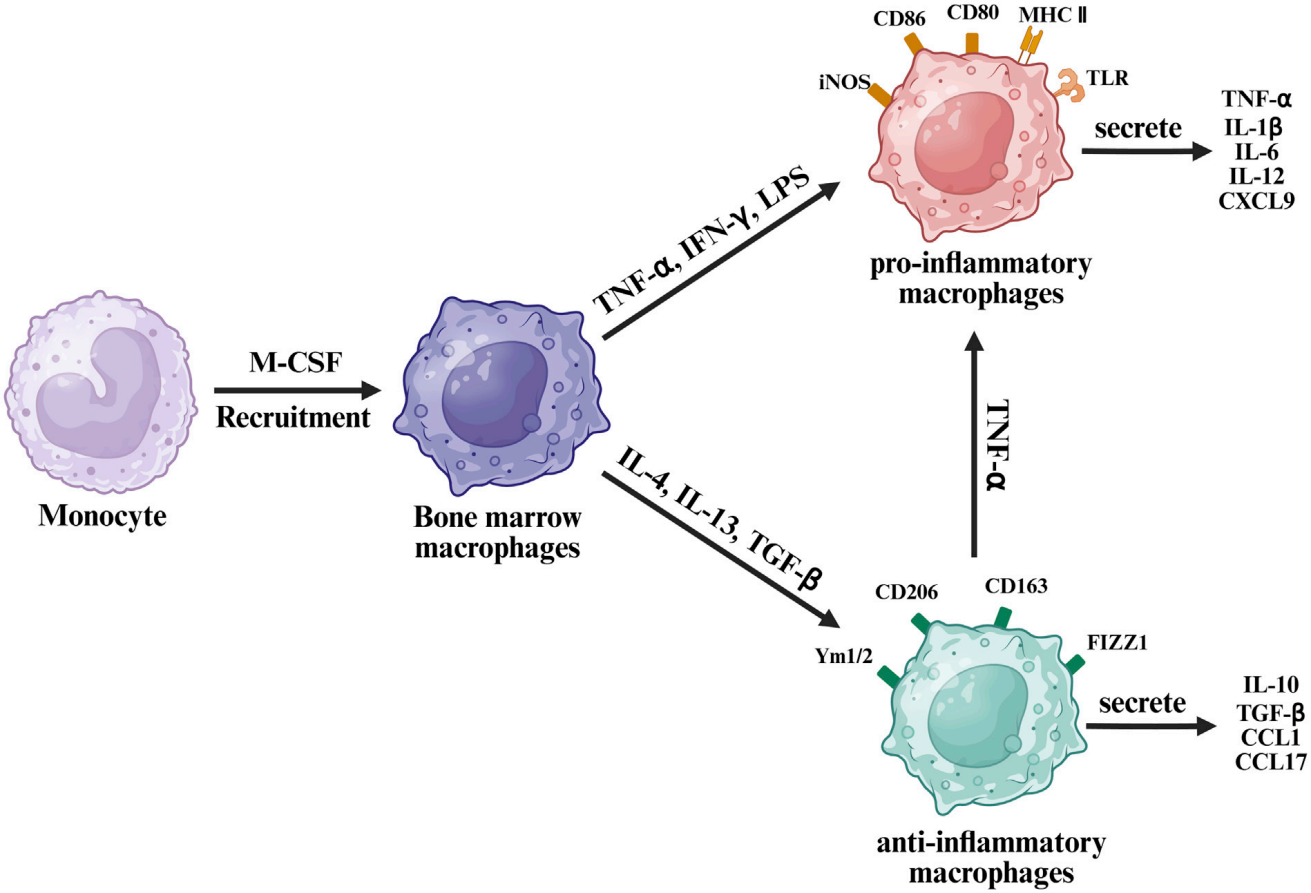
Schematic diagram of macrophage polarization from monocytes under different cytokine stimuli.

Upon fracture, most recruited macrophages are derived from bone marrow monocytes, termed M0 macrophages, rather than resident osteomacs (Perdiguero and Geissmann, 2016). These precursor cells differentiate under the influence of macrophage colony-stimulating factor (M-CSF), lipopolysaccharides (LPS), and interferon-gamma (IFN-γ) (Bosco, 2019). Subsequently, they can polarize into two major phenotypes: pro-inflammatory M1 and anti-inflammatory M2 phenotype macrophages (Yunna et al., 2020). This polarization is highly responsive to local environmental cues—M1 polarization is driven by stimuli such as LPS and helper T1 (Th1) cytokines, whereas M2 polarization is induced by cytokines including interleukin-4 (IL-4), IL-10, IL-13, IL-33, and transforming growth factor-beta (TGF-β) (Figure 2) (Bosco, 2019).

The M2 macrophage phenotype can be divided into four phenotypes based on function and stimuli. M2a macrophages are induced by IL-4 or IL-13, M2b by immune complexes and LPS, showing high chemokine ligand 1 (CCL1) and tumor necrosis factor alpha (TNF-α) expression, M2c by glucocorticoids or IL-10, and M2d by adenosines or IL-6 (Huang et al., 2018). M2a macrophages are primarily associated with anti-inflammatory responses and bone regeneration (Qiao et al., 2023). M2b macrophages represent an intermediate phenotype, modulating inflammation by secreting both pro-inflammatory and anti-inflammatory cytokines, with notably low IL-12 production (Wang et al., 2019; Yue et al., 2017). The M2c phenotype exhibits anti-inflammatory characteristics comparable to those of M2a macrophages; however, its gene expression peaks significantly earlier post-injury, approximately 6 h, compared to around 25 days for M2a (Lurier et al., 2017). M2d macrophages are typically involved in immunosuppression and angiogenesis within tumor microenvironments (Tamura et al., 2018) and chronic wounds through production of proangiogenic factors like vascular endothelial growth factor (VEGF) (Lai et al., 2019). Nonetheless, their precise roles in fracture healing remain controversial (Wermuth and Jimenez, 2015). Due to this functional diversity and complexity, this review simplifies these phenotypes collectively as “M2” for clarity.

M1 macrophages are characterized by expression of surface markers such as toll-like receptors, CD80, CD86, inducible nitric oxide synthase, and major histocompatibility complex class II, serving as hallmark indicators. Conversely, M2 macrophages express distinct markers, including CD206, CD163, CD209, FIZZ1, and Ym1/2^27^. Moreover, transcription factors differ significantly between these phenotypes. M1 macrophages secrete pro-inflammatory cytokines, such as TNF-α, IL-1β, IL-6, IL-12, and C-X-C motif chemokine ligand 9 (CXCL9). M2 macrophages, in contrast, secrete anti-inflammatory molecules, such as IL-10, TGF-β, CCL1, and CCL17 (McCauley et al., 2020).

Interestingly, under specific microenvironmental conditions, M1 and M2 macrophages are not fixed terminal states but demonstrate significant polarization plasticity—the capacity to switch between phenotypes in response to environmental cues reversibly. For instance, exposure to pro-inflammatory cytokines (such as TNF-α) can cause M2 phenotype macrophages activated by macrophage CSF to transform into a cell state with M1 phenotype characteristics (Zhao et al., 2015). This dynamic adaptability allows macrophages to adjust their function according to the evolving immune microenvironment: M1 phenotype macrophages generally mediate pro-inflammatory and antimicrobial responses, whereas M2 phenotype macrophages are associated with anti-inflammatory effects and bone regeneration (Shapouri-Moghaddam et al., 2018).

Clinically, fractures are a common form of bone injury (Dare and Hu, 2017). Following fracture, a rapid inflammatory response mediated by M1 macrophages occurs, subsequently transitioning toward osteoblast recruitment and bone regeneration facilitated by M2 macrophages. Both M1 and M2 phenotype macrophages are integral to osteogenesis. Loi et al. showed that the co-culture of macrophages with osteoblasts significantly enhanced osteogenic gene expression and matrix mineralization (Loi et al., 2016). However, prematurely polarized M2 phenotype macrophages may produce excessive fibrotic cytokines, promoting fibrous capsule formation around scaffolds and negatively impacting implant integration (Wynn and Vannella, 2016).

Furthermore, the phenotype of macrophages is not isolated but represents a dynamic equilibrium state. Usually, a relatively high M1/M2 ratio appears immediately after the bone injury, and then gradually transitions to a state dominated by M2 phenotype macrophages in the further stage of bone regeneration progress (Schlundt et al., 2018). This dynamic interplay highlights the complexity and multifaceted roles of macrophages in bone regeneration progress, emphasizing the necessity of a precise understanding of the dynamic polarization mechanism of macrophages for promoting bone tissue regeneration strategies.

While the M1/M2 classification offers a useful framework, it oversimplifies the real-time complexity of macrophage phenotypes observed *in vivo*. In fact, macrophages are often in an intermediate or mixed state, and their polarization is influenced by a continuum of signals, including cytokine gradients, matrix stiffness, metabolic conditions, and cellular crosstalk. The current classification methods based on markers cannot accurately capture this heterogeneity. Moreover, most mechanistic studies are performed *in vitro* using extreme polarizing conditions (such as LPS or IL-4), which may not reflect physiological states in bone injury.

Future research should integrate single-cell transcriptomics, spatial immune profiling, and dynamic imaging to better characterize macrophage subpopulations during bone regeneration. This nuanced understanding will be essential to harness macrophage polarization as a therapeutic target in regenerative medicine.

### 2.2 Macrophages in bone homeostasis

Osteomacs have garnered growing attention for their essential role in maintaining skeletal homeostasis. The pioneering work of Hume and colleagues in 1984 was among the first to identify osteomacs as tissue-resident macrophages definitively (Hume et al., 1984). Using immunohistochemical staining for F4/80 in sagittal sections of murine long bones, they demonstrated the widespread presence of F4/80^+^ mature macrophages distributed along cortical endosteal and periosteal surfaces, as well as the bone-lining tissues of trabecular bone (Chang et al., 2008). These cells were also frequently observed in perivascular regions, suggesting a close anatomical and functional association with vascular networks.

Osteomacs tend to localize near sites of bone formation and are often observed forming canopy-like structures with overactive cuboidal osteoblasts. Vi et al. further elucidated their relevance by demonstrating that osteomacs are involved in postnatal bone growth and the maintenance of bone mass (Vi et al., 2015). In a murine model with targeted deletion of lysozyme-M-expressing macrophages, progressive bone loss was observed by 3 months of age, accompanied by significant reductions in cortical bone mineral density and thickness. Interestingly, this model’s fetal long bone development remained unaffected, indicating that osteomacs are dispensable during embryogenesis but become essential for bone maintenance after birth.

These findings underscore the importance of osteomacs in bone remodeling, particularly in postnatal skeletal integrity. Their spatial proximity to osteoblasts, organization into canopy-like architectures, and presence in vascular niches suggest active roles in osteogenesis, potentially via coupling signals that coordinate bone formation and resorption.

However, despite these advances, the biology characteristics of osteomacs remains largely unclear. Particularly notable is that the molecular signals mediating communication between osteomacs and osteoblasts have not been fully elucidated. While paracrine factors are assumed to be involved, there is very limited evidence regarding the specific cytokines, membrane-bound ligands, or extracellular vesicles that may facilitate this interaction. In addition, it remains unclear whether osteomacs derive exclusively from embryonic progenitors or can be replenished by circulating monocytes under physiological or pathological conditions.

### 2.3 Macrophages in intramembranous ossification

Intramembranous ossification is a direct form of bone formation that occurs under stable fixation and minimal interfragmentary movement, whereby mesenchymal progenitors differentiate into osteoblasts without a cartilage intermediate (Schmidt-Bleek et al., 2014).

In a pioneering study, Alexander et al. (Alexander et al., 2011) demonstrated for the first time that macrophages are actively involved in this process. Using a murine tibial bone injury model, they identified F4/80^+^ osteomacs persisting throughout the healing period. Depletion of macrophages using the MaFIA (macrophage Fas-induced apoptosis) transgenic system (Burnett et al., 2004) or clodronate liposomes (Rooijen and Kesteren-Hendrikx, 2003) at the time of surgery led to markedly impaired woven bone deposition and mineralization. When the removal of macrophages was carried out on the third day after the injury, similar damage was observed. This indicates that macrophages are particularly important in the early stage of bone repair progress.

Further dissecting the role of specific macrophage subsets, (Batoon et al., 2019) used CD169-DTR mice to selectively deplete CD169^+^ osteomacs. Diphtheria toxin administration effectively reduced osteomac populations without affecting osteoclasts or trabecular bone mass. This depletion was accompanied by a substantial decline in osteoblast numbers, highlighting a supportive, non-redundant role of osteomacs in sustaining osteoblast viability and function.

In addition to direct cell–cell interactions, cytokine signaling appears to mediate macrophage-osteoblast crosstalk. Guihard et al. identified oncostatin M (OSM), a macrophage-derived cytokine, as a key contributor to intramembranous bone healing (Guihard et al., 2015). Compared with the wild-type control group, the OSM gene knockout mice showed a delay in bone formation in the early stage of the model establishment, but by the 14th day, their overall bone mass had returned to normal. These findings imply that OSM may be essential for initiating osteogenesis but less critical during the remodeling phase.

Despite the compelling evidence *in vivo*, the functional characteristics and mechanisms of osteomacs during the intracellular bone formation process remain largely unclear. While the anatomical proximity of osteomacs to osteoblasts and their depletion-linked osteogenic deficits suggest a supportive role, the precise molecular mediators—whether cytokines, exosomes, or juxtacrine signals—have not been comprehensively elucidated. Moreover, it is unclear whether these macrophages possess distinct activation or polarization states or whether their function is context dependent.

Although osteomacs have demonstrated essential roles in intramembranous bone formation, their molecular functions remain incompletely elucidated. It is still unclear whether they exert their effects through specific cytokines, extracellular vesicles, or direct juxtacrine interactions. Moreover, the polarization status of osteomacs remains to be defined. A particularly underexplored area is their response to mechanical cues, such as strain or matrix stiffness, which are inherent to the bone environment. Future investigations should focus on characterizing osteomac behavior under physiologically relevant mechanical conditions to better understand their regulatory functions in early bone regeneration.

### 2.4 Macrophages in endochondral ossification

**FIGURE 3:**
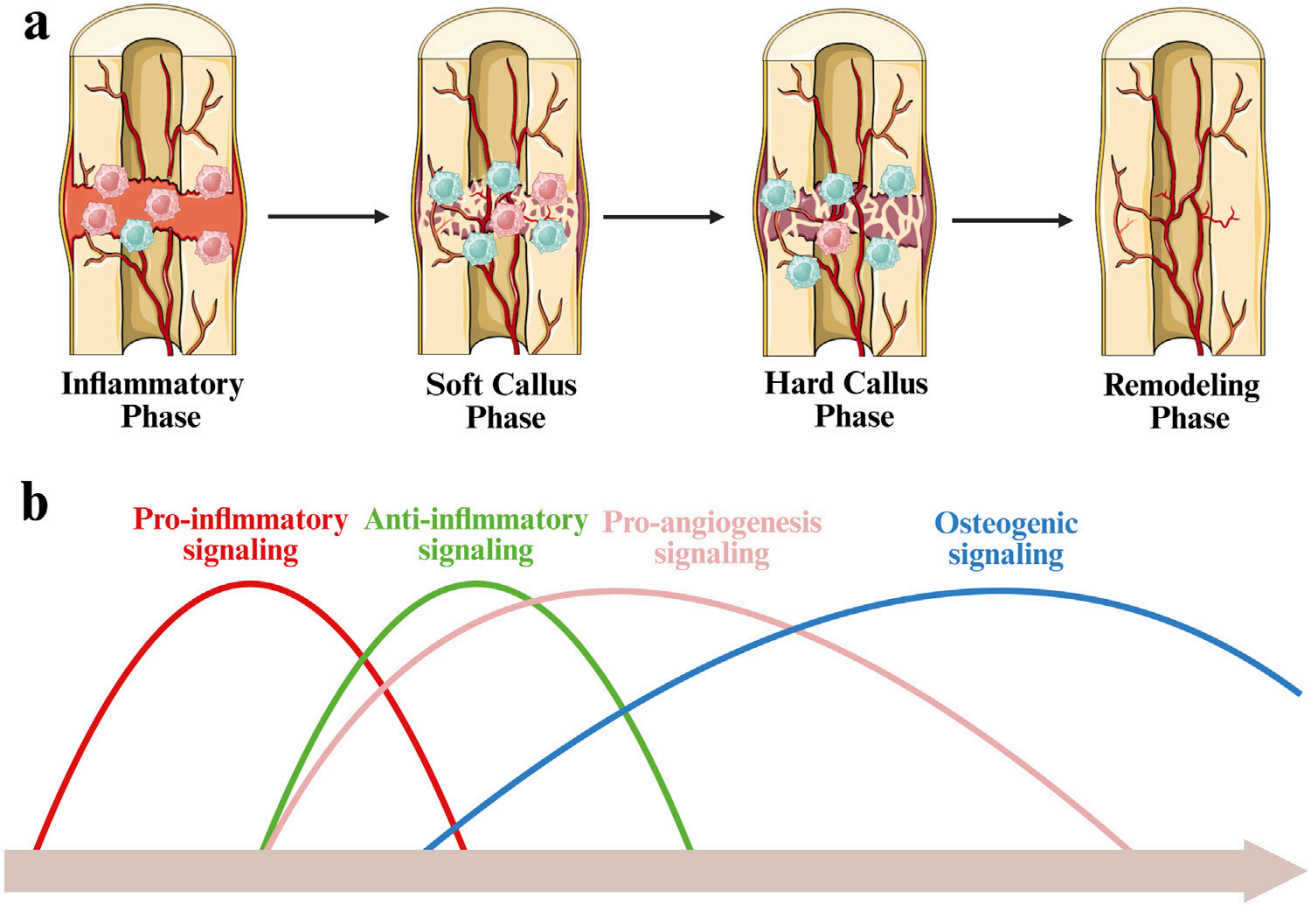
Schematic diagram of immunological regulation during bone fracture healing. **(a)** During the inflammatory phase, M1 macrophages are predominant. As healing progresses into the soft and hard callus stages, M2 macrophages increase and become dominant. **(b)** Timeline of bone fracture healing, illustrating the sequential and overlapping activation of key biological signals.

Endochondral ossification is the predominant mode of bone regeneration in clinical cases where fracture ends are unstable or separated by persistent gaps. This regenerative process is classically divided into four temporally overlapping phases: an initial inflammatory response, soft callus formation, hard callus formation, and final bone remodeling. M1 phenotype macrophages are predominantly present at the fracture site during the early inflammatory phase. As bone regeneration progress into the callus formation stages, the number of M1 phenotype macrophages gradually declines, while M2 phenotype macrophages increase and become the dominant phenotype, contributing to bone tissue repair and regeneration progress (Figure 3a) (Gou et al., 2024).

Bone repair and regeneration is governed by a series of temporally coordinated signaling events, including inflammation, angiogenesis, and osteogensis. The process is initiated by a sharp increase in pro-inflammatory signaling at the early stage of inflammation. As the regeneration progress to the cartilage callus stage, anti-inflammatory signaling gradually dominates, which helps eliminate inflammation and promote tissue stability. Concurrently, pro-angiogenic signaling rises, facilitating neovascularization within the fracture microenvironment. This vascularization is crucial for providing oxygen and nutrients and supporting the transition to the hard callus stage. Bone formation signals continue to increase steadily and reach their peak in the hard callus and remodeling stages, thereby coordinating the deposition of bone matrix and structural restoration (Figure 3b) (Schlundt et al., 2018; Pajarinen et al., 2019).

A hallmark distinguishing endochondral from intramembranous ossification is the formation of a cartilaginous callus. In a seminal study, Raggatt et al. demonstrated that depletion of macrophages abolished callus formation and bone regeneration in a murine femoral fracture model (Raggatt et al., 2014). Using the MaFIA (macrophage Fas-induced apoptosis) transgenic system, they showed that macrophage ablation at the time of injury completely inhibited new bone formation. When depletion was delayed to the granulation phase, mice developed smaller, fibrotic soft calluses compared to controls. Immunohistochemical analysis revealed that F4/80^+^Mac-2^-^ osteomacs primarily populated the maturing hard callus, while inflammatory F4/80^+^Mac-2^+^ macrophages were localized within early chondrification centers and persisted at the periphery of the expanding soft callus.

Consistent with these research results above, Schlundt et al. reported that macrophage depletion via clodronate liposomes at early healing stages led to the delayed formation of hard callus, smaller bone callus size, and impaired fracture union (Schlundt et al., 2018). Collectively, these studies emphasize the indispensable role of macrophages throughout the process of endochondral ossification, particularly during the period of inflammatory and soft callus phases.

Interestingly, the functions of macrophages have specific dependencies in different ossification patterns. During intramembranous ossification, they support woven bone deposition; endochondral ossification facilitates soft callus formation and progenitor cell recruitment (Wan et al., 2020). Despite these differing roles, a unifying theme is the early immunomodulatory function of macrophages in orchestrating local cell fate decisions and tissue transitions.

Despite extensive evidence supporting the critical involvement of macrophages throughout endochondral ossification, current experimental models exhibit significant limitations. The MaFIA transgenic system and clodronate liposome depletion both lack cell-type specificity. They may lead to off-target effects such as systemic immune dysregulation or unintended osteoclast depletion, thereby confounding the interpretation of bone regeneration phenotypes (Frith et al., 1997; Wu et al., 2017). Future studies should implement more targeted strategies to address these challenges, including inducible bone-specific Cre-lox systems or nanoparticle-mediated macrophage modulation. In parallel, resolving macrophage phenotypes’ spatial and temporal heterogeneity, particularly the distinction between inflammatory and reparative populations, will be essential for elucidating their dynamic roles in skeletal regeneration and for informing precision immunomodulatory therapies.

### 2.5 Macrophages in angiogenesis

Angiogenesis is an essential component of bone regeneration, providing oxygen and nutrients to support cellular proliferation, matrix deposition, and tissue remodeling. Notably, macrophages serve as a central source of pro-angiogenic factors during this process (Sunderkötter et al., 1991).

Jetten et al. reported that M2 macrophages exhibit increased expression of angiogenesis-related genes, including basic fibroblast growth factor, insulin-like growth factor-1, chemokine ligand 2, and placental growth factor (PlGF) (Jetten et al., 2014). Both IL-4-induced M2a and IL-10-induced M2c macrophages were shown to promote angiogenesis *in vitro* and *in vivo*. Mechanistically, M2c macrophage-driven angiogenesis appears to depend on PlGF signaling, whereas M2a-driven effects may be mediated via fibroblast growth factor pathways.

In addition to secreted cytokines, exosomes derived from M2 macrophages also facilitate angiogenesis. These extracellular vesicles carry a repertoire of reprogramming factors, cytokines, and growth factors that stimulate endothelial cell behavior (Huang et al., 2022; Kim et al., 2019). For instance, Lyu et al. demonstrated that M2 exosomes transfer miR-21 into human umbilical vein endothelial cells (HUVECs), where they inhibit PTEN and activate the AKT/mTOR signaling cascade, enhancing angiogenic activity (Yang et al., 2021).

While M1 macrophages are generally considered anti-angiogenic (Liu et al., 2020; Chen et al., 2021), some studies indicate that they may also contribute to angiogenesis under specific conditions. Guo et al. revealed that fatty acid-binding protein 4, secreted by M1-polarized macrophages, promotes angiogenesis and cartilage degradation in the context of rheumatoid arthritis (Guo et al., 2022), suggesting that M1-driven angiogenesis may be context-dependent and pathological.

Recognizing the temporal dynamics of macrophage polarization, Spiller et al. developed a sequential cytokine delivery strategy for scaffold-mediated bone regeneration (Spiller et al., 2015). Their system incorporated an early burst release of IFN-γ to induce M1 polarization, followed by a sustained release of IL-4 via biotin–streptavidin binding to promote a shift toward M2 polarization. This approach aimed to balance early inflammatory stimulation with subsequent tissue regeneration and neovascularization.

Despite growing evidence for macrophage-mediated angiogenesis, several mechanistic questions remain. First, while M2-derived factors such as PlGF have been implicated, the full range of angiogenic mediators and their precise temporal roles are not fully elucidated. It remains unclear whether specific M2 phenotypes play non-redundant or synergistic roles in orchestrating angiogenic cascades.

Moreover, the angiogenic potential of M1 macrophages may be underestimated in tissue-engineered contexts. Their ability to secrete VEGF, matrix metalloproteinases (MMPs), and pro-inflammatory chemokines may transiently facilitate early vascular sprouting, especially under hypoxic or ischemic conditions. The prevailing binary classification of M1/M2 phenotypes may thus oversimplify the polarization and functional spectrum of macrophages in neovascularization.

Future studies should employ single-cell spatial profiling, *in vivo* imaging of neovascular dynamics, and controlled microenvironmental manipulations to disentangle macrophage subset-specific contributions to bone-associated angiogenesis. Developing biomaterials that can temporally cue macrophage transitions in sync with vascular maturation will be key to enhancing regenerative outcomes.

### 2.6 Macrophages in neurogenesis

The interplay between the nervous system and the skeletal system has been increasingly recognized as an essential component of bone homeostasis and repair. Elefteriou et al. systematically reviewed the autonomic and sensory nervous system’s regulatory role in bone metabolism, noting that nerve injury or pharmacologic denervation significantly impairs bone mass and remodeling (Elefteriou, 2018). Peripheral sensory nerves innervating bone tissues release neuropeptides such as calcitonin gene-related peptide (CGRP) and substance P, which regulate osteoblastic activity, angiogenesis, and the local immune microenvironment (Duan et al., 2017; Lim et al., 2017). These neuropeptides not only act directly on osteogenic cells but also exert immune-modulatory effects, particularly on macrophages, thus influencing the entire regenerative milieu.

More recent experimental studies have extended this understanding into the domain of regenerative medicine. For instance, Wang et al. (2025) developed a sequential releasing hydrogel, showing that activation of local sensory nerves via CGRP not only enhances bone repair in osteoporotic models but also correlates with an immunoregulatory shift in the injury niche. However, the study did not elucidate whether CGRP-induced bone regeneration was mediated through direct neuro-osteogenic signaling or via immune cell intermediates, particularly macrophages. Cui et al. (2025) provided another compelling example, where Schwann cell-derived exosomes were shown to simultaneously promote osteogenesis, angiogenesis, and neurogenesis during periodontal bone regeneration. While the authors demonstrated beneficial effects on bone healing, the mechanistic involvement of immune cells such as macrophages remained speculative. This highlights a recurring limitation in the literature: although neurogenic stimuli are acknowledged as regenerative cues, the precise immune intermediates that integrate neural and skeletal responses are often underexplored.

Moreover, neural stem/progenitor cells (NSPCs), traditionally studied in neuroregeneration, have shown potential in bone healing when co-administered with osteoconductive scaffolds. Muangsanit et al. (2025) emphasized that the regenerative efficacy of NSPCs is significantly enhanced in anti-inflammatory environments, implying that macrophage polarization may be a critical determinant of their fate and function in bone-related contexts.

In addition to biochemical signals, biophysical properties of biomaterials—particularly electrical conductivity—have emerged as critical regulators of the immune-neural interface. Conductive scaffolds not only provide a permissive environment for neuronal signal transduction but also influence the polarization state of local macrophages. For example, Cheng et al. engineered a scaffold capable of delivering electrical stimulation to injured sacral nerves (Cheng et al., 2024). Their study demonstrated that the combination of conductive material and ES not only restored axonal continuity and remyelination to levels comparable with autografts but also significantly promoted M2 macrophage polarization. Importantly, while the study was conducted in a peripheral nerve model, the dual modulation of macrophages and neurons suggests translational potential for bone-related applications, particularly in cases where neural innervation is integral to skeletal regeneration.

Taken together, current evidence supports a model in which biomaterial-derived physical and biochemical cues shape macrophage polarization, which in turn modulates neurogenesis and consequently enhances bone regeneration. However, this causal cascade is often inferred rather than demonstrated, and many studies do not clearly separate direct neurogenic effects from immune-mediated ones.

### 2.7 Macrophages recruitment

The recruitment of immune cells, particularly macrophages, to injured or inflamed sites is a tightly regulated multistep process that involves tethering to the vascular endothelium, rolling, firm adhesion, intraluminal crawling, and ultimately transmigration through the endothelial barrier. Each of these steps is mediated by distinct sets of integrins and selectins, which are activated in response to inflammatory cues. Recent proposals have suggested harnessing this adhesion cascade through integrin agonists to enhance macrophage recruitment at sites requiring regeneration (Argyle and Kitamura, 2018).

In pathological or regenerative contexts, additional macrophages are recruited to local tissues in addition to the resident macrophage population. These recruited cells then undergo polarization toward M1 or M2 phenotypes in response to environmental stimuli such as cytokines, matrix stiffness, and metabolic signals (Wang et al., 2021a). This process allows macrophages to adopt functionally distinct roles across different phases of healing.

A central component regulating macrophage migration is the chemokine system. Chemokines are a family of over 50 small signaling proteins in humans and mice, grouped into four subfamilies based on cysteine residue positioning (Schall and Proudfoot, 2011). These chemokine–receptor pairs orchestrate the trafficking, homing, and retention of macrophages and other immune cells across tissues. Notably, many chemokines exhibit redundancy and promiscuity, with some ligands binding multiple receptors and *vice versa*, yet the affinities and functional consequences of these interactions can vary markedly.

As a practical application of this principle, Yang et al. developed a biomimetic peptide hydrogel scaffold functionalized with CX3CL1, which significantly enhanced the recruitment of circulating M2-like macrophages to the site of injury (Yang P. et al., 2023). This strategy offers a promising direction for the targeted modulation of the immune microenvironment during bone healing.

Although significant progress has been made in understanding macrophage recruitment, several mechanistic gaps remain. First, most studies emphasize ligand–receptor chemotaxis but overlook the role of biophysical parameters such as interstitial flow, matrix porosity, and adhesion ligand density, which can substantially modulate immune cell infiltration. Second, redundancy within the chemokine system complicates targeted modulation efforts. The simultaneous expression of multiple chemokines and receptors within damaged tissues raises concerns about off-target recruitment or dysfunctional polarization.

Furthermore, many scaffold-based delivery systems that incorporate chemokines do not mimic the endogenous spatiotemporal gradients necessary for physiological cell trafficking. Sustained or uncontrolled chemokine release may lead to excessive infiltration or prolonged inflammation, ultimately impairing tissue repair.

Therefore, future designs should integrate smart biomaterials capable of releasing chemokines in a spatiotemporally controlled manner, possibly in response to endogenous inflammatory markers or environmental cues.

## 3 Physical cues from biomaterials modulate macrophage polarization

### 3.1 Stiffness

Biomaterial stiffness has emerged as a critical biophysical cue influencing cell fate and function during bone regeneration. Defined by Young’s modulus (the ratio of stress to strain), stiffness directly shapes cellular behaviors such as adhesion, migration, and phenotype commitment through mechano-transduction signaling. In the context of macrophages, this physical parameter not only affects immune responses but also modulates their polarization states, thereby influencing the inflammatory environment and subsequent tissue healing.

**FIGURE 4:**
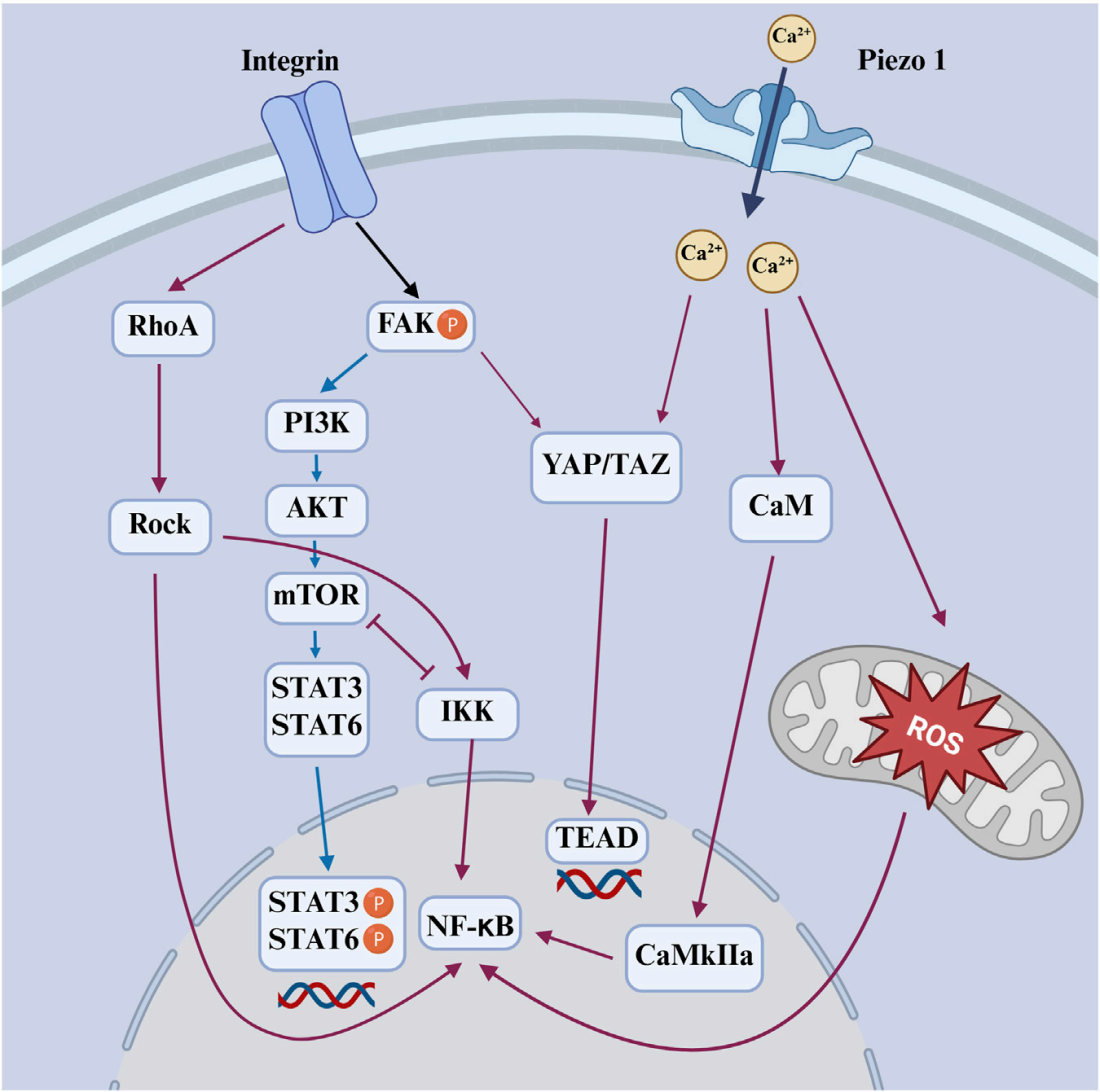
Schematic diagram of mechanical signals sensed by integrin and Piezo1 activate downstream pathways that regulate macrophage polarization. Red-orange arrows indicate M1-promoting pathways; blue arrows indicate M2-promoting pathways.

Macrophages utilize a diverse repertoire of mechanosensitive systems to decode the physical properties of their microenvironment and translate them into functional responses. This process, known as mechanotransduction, involves the activation of surface receptors, ion channels, and cytoskeletal elements in response to mechanical cues from the extracellular matrix. Key components include integrin-based adhesion complexes, which couple extracellular stiffness to cytoskeletal tension; mechanosensitive ion channels, such as Piezo1 (Atcha et al., 2021), which convert membrane stretch into calcium-dependent signaling cascades; and cytoskeletal structures like podosomes and filopodia, which dynamically probe substrate topography and stiffness. Furthermore, nuclear mechanotransduction pathways and transcriptional regulators such as YAP/TAZ integrate these signals to modulate gene expression, thereby influencing macrophage polarization, inflammatory signaling, and metabolic activity (Figure 4). Notably, YAP/TAZ plays a context-dependent role, contributing to either M1 or M2 polarization based on microenvironmental cues. Collectively, these interconnected modalities coordinate immune cell behavior and adaptation across both homeostatic and pathological tissue environments (Lee et al., 2022).

Recent studies have demonstrated that matrix stiffness promotes macrophage M1 polarization through the Piezo1–YAP signaling axis, where mechanical cues induce calcium influx and YAP nuclear translocation to drive pro-inflammatory gene expression (Figure 4) (Mei et al., 2024). Consistently, YAP-mediated mechanotransduction has been shown to fine-tune macrophage inflammatory responses by modulating cytokine production and metabolic programming, highlighting YAP as a central node linking mechanical stimuli to immune activation (Meli et al., 2020).

Mechanistically, podosomes function as specialized actin-rich mechanosensory structures unique to macrophages and other myeloid cells. Atomic force microscopy studies have quantified podosome stiffness to be approximately 40 kPa, exhibiting dynamic cyclic variations linked to actomyosin contractility (Labernadie et al., 2010). These structures act as localized sensors and transducers of extracellular mechanical signals, enabling macrophages to probe substrate rigidity and adapt their adhesion and signaling accordingly (Linder and Wiesner, 2016).

Complementary to podosomes, macrophage filopodia—slender, finger-like protrusions composed of bundled actin filaments—play a critical role in exploring the extracellular matrix and detecting nanoscale mechanical cues. Recent optical tweezer-based experiments demonstrated that filopodia display adaptive mechanotransduction properties characterized by cycles of force-induced rupture and reformation, akin to ‘catch-bond’ behavior, enabling sustained mechanical sensing and signaling under variable tensile forces (Michiels et al., 2022).

Moreover, filopodia contribute to intercellular communication by releasing cytokine-containing vesicles at their tips, which may modulate local inflammatory environments and further influence macrophage functional states (Zhu et al., 2023). Together, podosomes and filopodia constitute an integrated mechanosensory toolkit that allows macrophages to finely decode and respond to biomaterial stiffness, thus coordinating their polarization, migration, and immunomodulatory functions in bone regeneration contexts.

Chen et al. engineered polyacrylamide hydrogel substrates with tunable stiffnesses—2.55 ± 0.32 kPa (soft), 34.88 ± 4.22 kPa (medium), and 63.53 ± 5.65 kPa (stiff)—mimicking the biomechanical range of collagen fibers, osteoid, and early calcifying bone, respectively (Chen et al., 2020). Their findings demonstrated that murine bone marrow-derived macrophages (BMDMs) cultured on softer substrates preferentially polarized toward the pro-inflammatory M1 phenotype, while intermediate stiffness favored anti-inflammatory M2 polarization. This transition was shown to involve modulation of the ROS-mediated NF-κB pathway, implicating a mechanosensitive regulatory mechanism in immune cell behavior (Figure 4).

However, the relationship between stiffness and macrophage phenotype is not unequivocal. Contradictory findings have been reported, especially in studies using gelatin methacryloyl hydrogels. Several reports observed that increasing stiffness promoted a greater proportion of M1 macrophages and heightened inflammatory responses (Zhuang et al., 2020; Li and Bratlie, 2021; Yuan et al., 2021), suggesting a context-dependent effect potentially influenced by differences in material composition, crosslinking density, and biochemical cues.

One of the key challenges in interpreting stiffness-related data is the confounding influence of other scaffold parameters. Stiffness rarely exists in isolation; it is often accompanied by changes in porosity, pore size, and matrix density, making it difficult to dissect its independent contribution. Addressing this, Jiang et al. developed a cryoprotectant-based system that decouples stiffness from pore size (Jiang et al., 2019). Their findings revealed that smaller, softer pores favored M1 polarization, whereas larger, stiffer pores promoted M2 phenotypes—apparently contradicting previous conclusions and underscoring the complex interplay between structural architecture and immune modulation.

Further complicating this landscape, Sridharan et al. demonstrated that the impact of scaffold stiffness on macrophage response is modulated by the crosslinking chemistry used during scaffold fabrication (Jiang et al., 2019). Using physical (dehydrothermal treatment) and chemical (genipin) crosslinkers on collagen scaffolds, they found that macrophage phenotypes varied not only with stiffness but also with the type of crosslinking agent, highlighting the multifactorial nature of material-immune interactions.

Beyond polarization, stiffness also governs macrophage motility modes. Sridharan et al. reported that on soft (11 kPa) and intermediate (88 kPa) substrates, macrophages exhibited rapid amoeboid migration driven by RhoA kinase (ROCK) but independent of podosome formation. In contrast, on stiff (323 kPa) substrates, macrophages adopted a slower, mesenchymal-like migration reliant on podosomes but independent of ROCK activity (Figure 4) (Sridharan et al., 2019). These results suggest that substrate stiffness not only tunes inflammatory phenotype but also reprogram macrophage locomotion strategies, which may influence their spatial dynamics within the regenerating tissue. Collectively, these findings underscore the nuanced and often non-linear role of scaffold stiffness in modulating macrophage behavior. Rather than serving as a singular determinant, stiffness interacts with other physical and biochemical cues of the scaffold to guide immune responses. Future scaffold designs must therefore consider not only the mechanical environment but also its integration with structural and chemical features to achieve precise immunomodulation for enhanced bone regeneration outcomes.

### 3.2 Viscoelasticity

While stiffness has long been recognized as a key mechanical cue in biomaterial design, emerging evidence underscores the critical role of viscoelasticity—the time-dependent deformation behavior of materials—in shaping immune cell responses, particularly macrophage polarization. Viscoelastic materials exhibit both solid-like (elastic) and fluid-like (viscous) behavior, more accurately mimicking the dynamic and heterogeneous extracellular matrix (ECM) environments encountered during tissue injury, inflammation, and remodeling (Chaudhuri et al., 2020).

Although early investigations into matrix viscoelasticity primarily centered on stem cell responses—for example, Chaudhuri et al. demonstrated that stress relaxation in alginate hydrogels modulates mesenchymal stem cell (MSC) fate and promotes osteogenic differentiation (Chaudhuri et al., 2016).

Recent studies have extended these principles to immune cells, demonstrating that the viscoelastic properties of hydrogels and other biomaterials significantly influence macrophage phenotype. For example, Liu et al. developed a liquid crystalline matrix with tailored viscoelastic properties to investigate its impact on macrophage behavior (Liu L. et al., 2023). Their study revealed that substrates with enhanced viscoelastic damping promoted a shift toward an anti-inflammatory M2 phenotype. Mechanistically, the modulation of cytoskeletal organization and focal adhesion formation was implicated in the macrophage response. Expanding on these findings, Kalashnikov and Moraes showed that substrate viscoelasticity modulates macrophage morphology and phagocytic capacity. Human macrophages on more viscous substrates displayed elongated shapes and greater phagocytosis, indicating enhanced M2-like functionality. These morphological adaptations were accompanied by reorganization of the actin cytoskeleton and changes in integrin engagement, suggesting that viscoelasticity regulates immune function via mechano-responsive structural remodelin (Kalashnikov and Moraes, 2023).

Moreover, viscoelasticity influences cell spreading, cytoskeletal organization, and focal adhesion dynamics, all of which are known upstream regulators of macrophage phenotype. On highly elastic substrates, macrophages tend to display a rounded morphology with strong actin stress fibers and NF-κB activation, favoring M1 polarization. In contrast, on viscoelastic materials that allow matrix deformation over time, macrophages adopt a more spread morphology, reduced cytoskeletal tension, and increased STAT6 activation, which are hallmarks of M2-like behavior (Figure 4) (Fang et al., 2025).

Importantly, matrix viscoelasticity not only shapes cytoskeletal architecture but also influences macrophage-mediated osteogenesis through metabolic reprogramming. Tao et al. reported that macrophages on viscoelastic substrates undergo a mechanotransduction-mediated metabolic switch from glycolysis toward oxidative phosphorylation, promoting an M2 phenotype that supports osteogenic differentiation of MSCs. This study highlights a tripartite coupling between viscoelastic cues, immunometabolism, and regenerative outcomes, reinforcing the significance of time-dependent matrix mechanics in bone healing (Tao et al., 2025).

Also, viscoelasticity modulates signaling duration and amplitude in response to inflammatory stimuli. Zhou and Wu revealed that soft substrates with tunable viscoelasticity amplify the temporal distinction between transient and sustained LPS-induced signaling. This finding suggests that macrophage sensitivity to inflammatory cues is contextually gated by the viscoelastic landscape, further supporting its role in immune fine-tuning (Zhou and Wu, 2022).

Together, these findings indicate that viscoelasticity regulates macrophage behavior through interconnected mechanical and biochemical pathways, including cytoskeletal dynamics, integrin signaling, transcriptional feedback, and metabolic plasticity. Integrating viscoelastic features into biomaterial design offers a promising strategy to orchestrate immunomodulation and promote constructive remodeling in bone regeneration.

### 3.3 Topography

Beyond biochemical cues, the geometric features of scaffolds, ranging from micro-to nanoscale, play a pivotal role in modulating macrophage behavior. Importantly, not only does scaffold topography influence cell fate, but the topography of the cell itself acts as a key determinant of macrophage phenotype and function.

Macrophage morphology has long been associated with their polarization state. M2 macrophages generally display an elongated, spindle-like shape, while M1 macrophages tend to exhibit a rounder morphology (Stöger et al., 2012; Chinetti-Gbaguidi et al., 2011). Intriguingly, studies employing micropatterning techniques to artificially constrain cell shape demonstrate that enforced elongation alone is sufficient to promote M2 marker expression and suppress pro-inflammatory cytokine secretion. This effect is abolished by inhibiting actin polymerization or actomyosin contractility, underscoring the role of cytoskeletal tension in topography-induced polarization (Folkman and Moscona, 1978). In a complementary manner, Tu et al. reported that cyclic mechanical stretch promotes pro-inflammatory macrophage activation through the RhoA–ROCK–NF-κB signaling axis, further emphasizing the importance of cytoskeletal dynamics in shaping macrophage functional states in response to physical forces (Figure 4) (Tu et al., 2022).

Interestingly, substrate stiffness also indirectly shapes macrophage topography. Macrophages cultured on stiffer substrates often spread and flatten, while those on softer surfaces tend to adopt a rounded shape (McWhorter et al., 2013; Blakney et al., 2012; Irwin et al., 2008). These morphological adaptations may partly explain the seemingly paradoxical M2-like polarization observed on certain high-stiffness materials. For example, Lin et al. demonstrated that co-culture with multi-walled carbon nanotubes promoted M2 polarization of macrophages, thereby enhancing osteogenesis of bone marrow mesenchymal stem cells (BMSCs) (Lin et al., 2023).

In the context of bone tissue engineering, cells are highly responsive to topographical cues embedded in scaffold surfaces. Hydroxyapatite nanoparticles (HANPs), widely utilized in bone regeneration, offer a compelling example. One study comparing four different HANP shapes (rods, dots, sheets, and fibers) found that fiber-shaped particles triggered the strongest immune response, as evidenced by elevated reactive oxygen species (ROS) generation in inflammatory cells (Pujari-Palmer et al., 2016). Though conducted in zebrafish embryos, these findings provide valuable insights into how nanoparticle morphology may regulate macrophage activation in mammalian systems. Micro/nano-sheet array structures have also been shown to modulate macrophage polarization by influencing cytoskeletal organization and focal adhesion dynamics. Such topographical cues promote M2-like phenotypes while suppressing inflammatory responses, likely through integrin-mediated activation of RhoA–ROCK and PI3K–Akt pathways. These effects contribute to a pro-regenerative immune environment favorable for osteogenesis (Figure 4) (Zheng et al., 2022).

**FIGURE 5:**
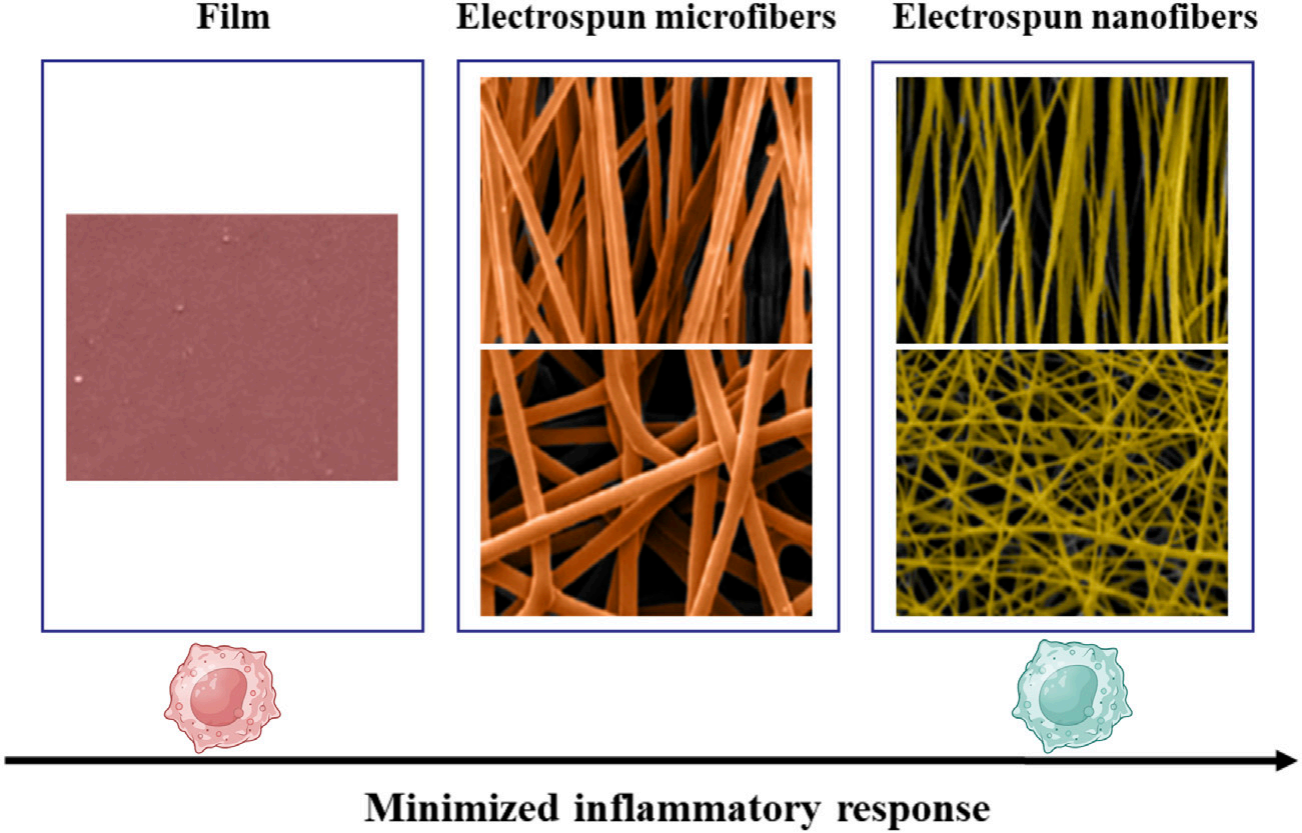
Inflammatory response of macrophages decreases from flat PLLA films to microfibers and further to nanofibers. Reprinted with permission from Saino et al. (2011). Copyright 2011 American Chemical society.

Electrospinning technology allows for the fabrication of fibrous scaffolds with precise alignment and diameter control. Saino et al. cultured macrophages on scaffolds composed of oriented polylactic acid (PLLA) microfibers (∼1.3 μm in diameter) and nanofibers (∼390 nm in diameter), in addition to flat PLLA films. Their results demonstrated that macrophages cultured on flat PLLA films exhibited the strongest pro-inflammatory response. While those on nanofibrous scaffolds displayed the lowest inflammatory activity. Interestingly, fiber alignment had relatively minor effects on inflammatory levels (Figure 5) (Saino et al., 2011).

At times, the surface topography of materials is described in terms of “roughness.” For example, titanium surfaces with mesoporous nano-cavitated structures (20–22 nm in diameter and 10–20 nm in depth) have been shown to modulate macrophage behavior. These surfaces produce topographic and chemical cues that drive macrophages toward a less inflammatory state (Ariganello et al., 2018). Furthermore, in a rat tibia implantation model, Karazisis et al. demonstrated that titanium implants modified with well-defined semispherical nanotopographies (∼75 nm) significantly reduced early inflammatory responses and enhanced early osteogenic activity through upregulation of osteocalcin expression and increased new bone formation within 3 days post-implantation (Karazisis et al., 2017). These findings provide *in vivo* confirmation that nanoscale topographies can orchestrate both immunomodulation and osteogenesis.

More complex geometries have also shown promise in immunomodulation. Carrara et al. developed biomimetic topographic substrates with hierarchical multiscale features, including periodic lattices and sub-micrometer cues (down to 500 nm), which promoted polarization toward the M2d macrophage phenotype. In other investigations, the topography of 3D features such as vertex angles (<60°) was found to critically affect cell adhesion and macrophage polarization. Sharp triangular pyramids, by contrast, dramatically reduced macrophage attachment and limited polarization altogether (Carrara et al., 2023).

Strikingly, these observations are parallel to biological systems. Lung epithelial tissues, for example, are notable for their intrinsic fractal geometries, reflecting a naturally evolved spatial organization that governs immune cell behavior (Vassey et al., 2023). By extension, the microstructure of fracture ends in bone may serve as a spatial cue for directing macrophage phenotype during regeneration.

In conclusion, these findings emphasize that scaffold topography is not merely a passive design feature but an active modulator of immune cell behavior. The spatial configuration—be it at the cellular, topographical, or architectural scale—can instruct macrophage polarization and function, ultimately influencing the regenerative outcome.

### 3.4 Pore size and porosity

Bone is inherently a porous tissue. Cancellous bone is composed of trabeculae with a porosity of 50%–90%, while cortical bone exhibits a much lower porosity, typically ranging from 5% to 10% (Cockerill et al., 2020; Maksimkin et al., 2017). Mimicking this structure, bone tissue engineering scaffolds are typically designed with 3D porous architectures that allow cellular infiltration, nutrient diffusion, and vascular ingrowth. Emerging evidence indicates that both pore size and overall porosity critically influence macrophage behavior, particularly polarization phenotypes.

**FIGURE 6:**
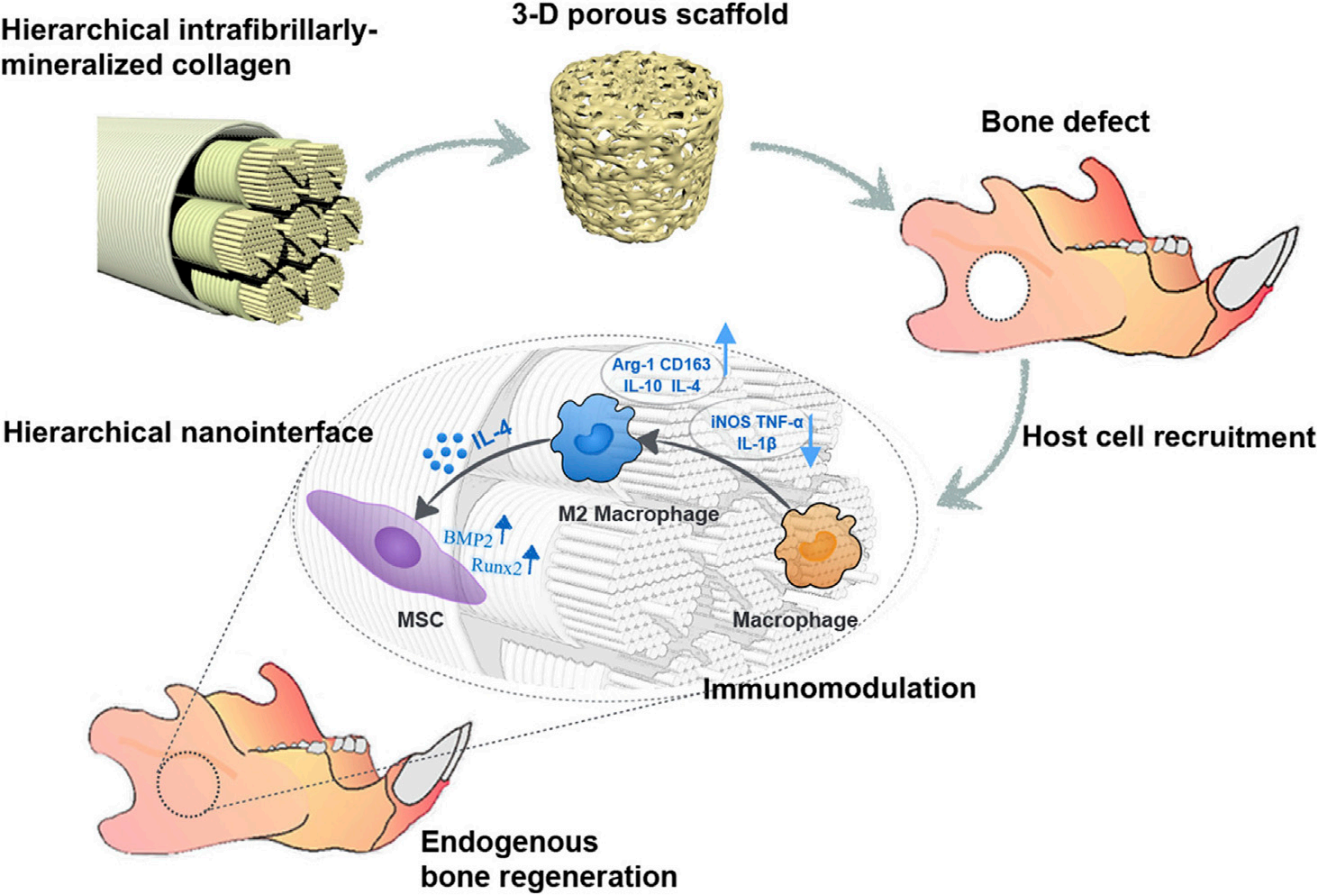
HIMC scaffolds with well-defined pore size and porosity promote endogenous bone regeneration by modulating macrophage polarization. Reprinted with permission Jin et al. (2019). Copyright 2019 American Chemical society.

To replicate the native bone microenvironment, scaffolds are increasingly fabricated with hierarchical porous structures. For instance, Jin et al. developed a hierarchical intrafibrillarly mineralized collagen (HIMC) membrane that mimics both the structural and compositional complexity of bone ECM. Although specific pore sizes were not examined, the staggered nanointerface and porous structure of the material promoted M2 macrophage polarization and enhanced host BMSC recruitment, thereby accelerating bone regeneration (Figure 6) (Jin et al., 2019). Similarly, Xuan et al. reported that HIMC membranes facilitate M2 polarization, which subsequently enhances BMSC migration and tissue integration (Xuan et al., 2022).

The role of pore confinement on macrophage phenotypes has been further explored using microporous annealed particle (MAP) scaffolds. Liu et al. examined MAP scaffolds composed of microgels with diameters of 40, 70, and 130 μm (Liu Y. et al., 2023). Their findings demonstrated that spatial confinement within pores of different scales elicited distinct morphological and phenotypic responses in macrophages. In 40 μm MAP scaffolds, macrophages were forced to elongate within tight pores and exhibited increased Arg1^+^CD206^+^ expression, indicating an anti-inflammatory M2-like state. By contrast, 70 μm scaffolds supported a more spherical cell and nuclear shape, associated with a mixed M1/M2 phenotype, which was also validated *in vivo*. Interestingly, in the 130 μm scaffolds, which allowed greater motility, macrophages displayed reduced CD11c expression despite low inflammatory markers, suggesting decreased antigen presentation capacity. These results highlight the critical role of physical confinement and cell shape in shaping macrophage fate within 3D environments.

Despite these insights, the underlying mechanisms remain incompletely understood. Yang et al. reported that scaffolds with larger pore sizes promoted M1-to-M2 transition, while smaller pores tended to entrap macrophages at the scaffold periphery, promoting an inflammatory M1 phenotype (Yang X. et al., 2023; Madden et al., 2010).

Beyond immunomodulation, pore architecture also regulates angiogenesis. Pore sizes that permit adequate oxygen diffusion support vascular ingrowth (Wang et al., 2021b; Meh et al., 2013), whereas excessively small pores may create hypoxic niches that trigger chronic inflammation (Kuboki et al., 2002). Hypoxia has been shown to drive macrophage metabolism toward glycolysis and pro-inflammatory activation, thereby exacerbating the inflammatory cascade and impairing bone repair (Díaz-Bulnes et al., 2020).

Nonetheless, increasing pore size and porosity often compromises mechanical strength. Thus, achieving an optimal balance between immunomodulatory capacity, angiogenic potential, and structural integrity remains a key challenge in scaffold design for bone regeneration.

### 3.5 Hydrophilicity

Once implanted into a bone defect, scaffolds are rapidly immersed in a dynamic immune microenvironment where their surface properties critically influence host responses. Among these properties, surface hydrophilicity has emerged as a key determinant of early macrophage behavior. Hydrophilic surfaces can modulate protein adsorption patterns, altering the biomolecular corona that cells first encounter. This, in turn, affects macrophage adhesion, morphology, and downstream polarization. Proteomic studies have shown that macrophages cultured on hydrophilic materials display distinct protein expression profiles and Calcitonin gene-related peptide exerts anti-inflammatory property through regulat altered cytokine and chemokine secretion, reflecting a shift in their functional phenotype (Dinnes et al., 2007).

Titanium, a commonly used orthopedic material, has served as a prototypical platform for studying hydrophilicity-induced immune modulation. Notably, modifications that increase titanium’s surface wettability—often through acid etching, UV treatment, or plasma activation—have been consistently associated with enhanced M2-like polarization. These surfaces upregulate anti-inflammatory mediators such as IL-4 and IL-10, while suppressing pro-inflammatory cytokines like TNF-α and IL-6 (Hotchkiss et al., 2016; Hamlet et al., 2019). In parallel, M2-associated gene markers are also elevated, indicating a functional immune shift toward a pro-healing state. These immunomodulatory effects have been linked to improved osteogenic signaling and enhanced osseointegration, highlighting the potential of surface chemistry as a design lever for next-generation immuno-instructive bone scaffolds.

Despite these promising outcomes, several limitations and unanswered questions remain. First, hydrophilicity does not operate in isolation. It is inherently intertwined with other surface attributes such as charge, topography, and stiffness, making it challenging to dissect its individual contribution. For example, increased hydrophilicity often co-occurs with enhanced surface energy and altered electrostatic interactions, both of which can independently affect protein adsorption and macrophage phenotype. Second, while *in vitro* studies often report robust M2 polarization on hydrophilic surfaces, the reproducibility and durability of these effects under dynamic *in vivo* conditions, which are characterized by fluid shear, protein exchange, and mechanical loading, are still poorly understood. Furthermore, the optimal range of hydrophilicity that balances immune regulation without inducing unintended fibrotic responses remains undefined.

### 3.6 Electromagnetic stimuli

Electromagnetic cues have been increasingly recognized as potent modulators of macrophage function and bone regeneration (Wosik et al., 2018; Xu et al., 2022). Given their dual role in modulating immunity and promoting osteogenesis, electroactive biomaterials have garnered increasing interest in the field of bone tissue engineering. Piezoelectricity, defined as the generation of electric potential upon mechanical deformation, has been observed in numerous biological tissues such as bone, cartilage, and tendons (Zengo et al., 1973). This phenomenon largely arises from the unique structural properties of the ECM, where collagen molecules, arranged in a non-centrosymmetric triple helix, constitute the primary source of biological piezoelectricity (Zhang et al., 2023).

**FIGURE 7:**
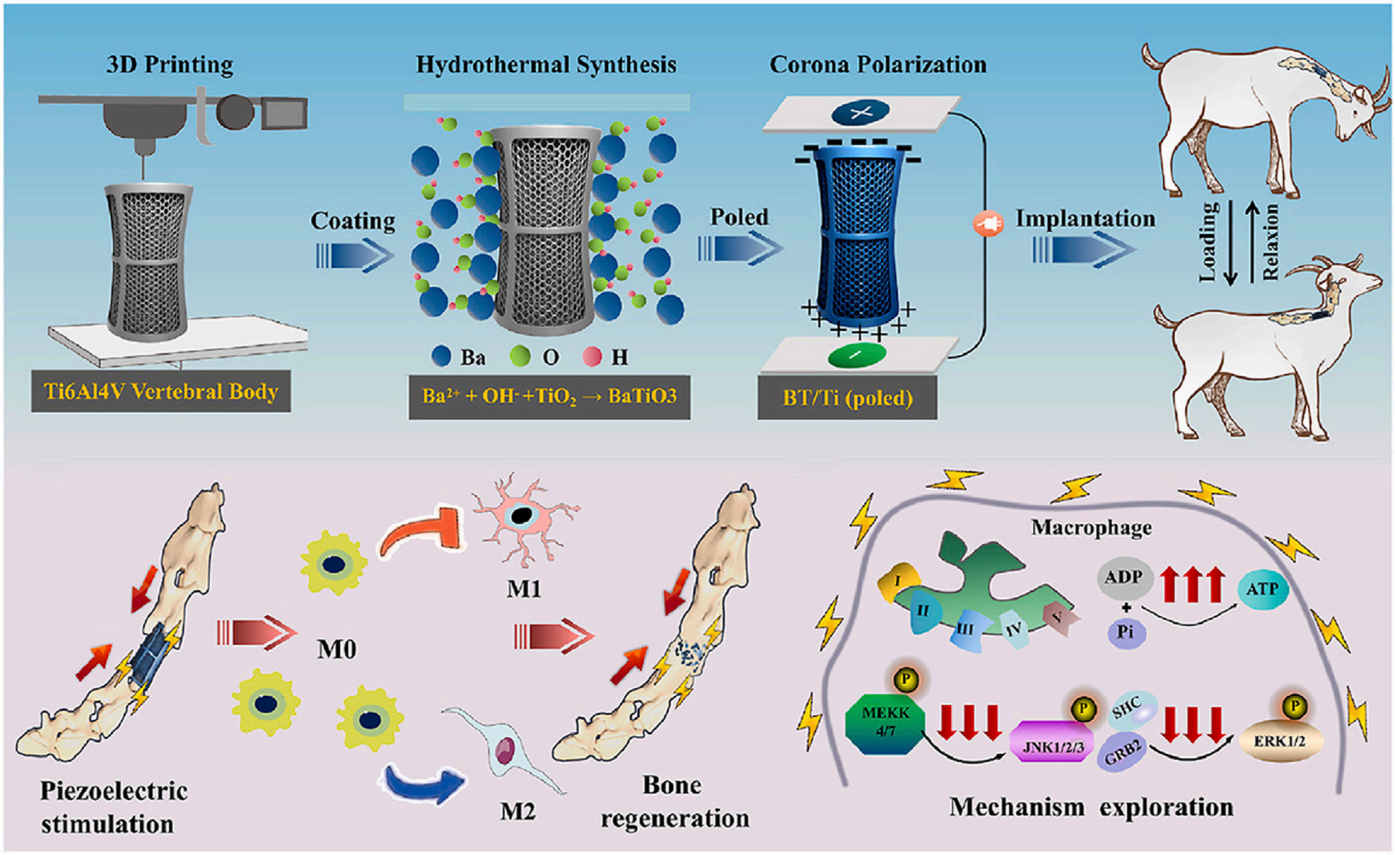
Piezoelectric BT/Ti scaffolds promote M2 macrophage polarization and enhance bone regeneration in a sheep cervical corpectomy model, accompanied by downregulation of MAPK/JNK signaling and upregulation of oxidative phosphorylation pathways. Reprinted with permission from Wu et al. (2023). Copyright 2022 Elsevier Ltd.

Recent advancements have leveraged these principles to design responsive scaffolds for immunomodulation. For example, Kong et al. developed a noninvasive strategy that utilizes ultrasound to stimulate piezoelectric β-PVDF films, generating localized electrical signals that promote calcium influx via voltage-gated channels (Kong et al., 2021). This process activates the Ca^2+^–CAMK2A–NF-κB pathway, resulting in selective M1 polarization and the release of pro-inflammatory chemokines. In contrast, piezoelectric materials such as barium titanate (BaTiO_3_) can generate electric signals in response to mechanical stress, thereby promoting apatite deposition, cell differentiation, and osteogenesis (Figure 4) (Ehterami et al., 2018; Tang et al., 2017). Wu et al. fabricated a piezoelectric BaTiO_3_/Ti_6_Al_4_V (BT/Ti) scaffold by hydrothermally synthesizing a uniform BaTiO_3_ layer on a 3D-printed titanium alloy base. They validated the regenerative potential of BT/Ti scaffolds in both *in vitro* and *in vivo* models. Following stimulation with low-intensity pulsed ultrasound, these scaffolds promoted M2 polarization of macrophages and enhanced osteogenesis in a sheep cervical corpectomy model. Transcriptomic analyses confirmed downregulation of MAPK/JNK signaling and upregulation of oxidative phosphorylation pathways, supporting the scaffold’s immunoregulatory function (Figure 7) (Wu et al., 2023). Altogether, these findings support an emerging strategy for improving bone healing through the design of bioelectrically active scaffolds.

Beyond material-driven effects, the parameters of electrical stimulation itself also influence macrophage behavior. Gu et al. demonstrated that a square waveform preferentially enhanced LPS/IFN-γ-induced M1 polarization, while a sinusoidal waveform promoted both M1 and IL-4-induced M2 polarization (Gu et al., 2022). In a related study, Hore et al. reported that electrical fields significantly enhanced macrophage phagocytic capacity toward various targets, including carboxylate beads, apoptotic neutrophils, and *Candida albicans* (Hoare et al., 2016).

However, despite the promising findings, several critical challenges remain in interpreting the role of electromagnetic cues in macrophage polarization. First, variations in experimental conditions, such as waveform parameters, frequency, field strength, and exposure duration, complicate direct comparison across studies and may lead to inconsistent polarization outcomes. Furthermore, most *in vitro* experiments are conducted in simplified systems that fail to recapitulate the dynamic mechanical and bioelectrical microenvironment encountered *in vivo*, where multiple signaling pathways converge.

Another limitation lies in the incomplete understanding of how macrophages sense and transduce electrical signals. While some studies have implicated voltage-gated calcium channels and downstream pathways such as Ca^2+^–CAMK2A–NF-κB, the upstream membrane receptors, mechanosensitive ion channels, and intracellular signaling crosstalk remain poorly defined. It is also unclear whether electrical stimuli act independently or synergistically with biochemical cues such as cytokines, matrix stiffness, or surface topography.

In addition, the influence of scaffold composition and architecture on local charge distribution is often overlooked. Piezoelectric effects may vary not only with material properties but also with microstructural anisotropy, degree of polarization, and the presence of insulating interfaces. Without standardized methods for characterizing and delivering electrical stimuli, reproducibility and clinical translation remain significant obstacles.

### 3.7 Metal composition

To regulate the local inflammatory microenvironment and promote bone regeneration, metallic elements such as magnesium, zinc, strontium, copper, and tantalum have been widely incorporated into scaffold designs (Chen et al., 2016).

Magnesium is notable for its rapid *in vivo* degradation into bioactive ions and its intrinsic anti-inflammatory properties (Bessa-Gonç et al., 2023; Seitz et al., 2014). Hu et al. demonstrated that Mg inhibits macrophage activation by downregulating both pro- and anti-inflammatory cytokine expression at the mRNA level, and by reducing IL-1β, IL-6, and IL-10 levels in the cell supernatant (Hu et al., 2018). Furthermore, Mg was shown to suppress the activation of nuclear factor-κB (NF-κB) by reducing its nuclear translocation and phosphorylation. Similarly, Cheng et al. reported that high-purity Mg screws inhibited M1 polarization and enhanced M2 polarization at the tendon–bone interface over a 4-week period (Chen et al., 2022).

Zinc, an essential trace element involved in immune regulation, is also widely applied in bone scaffolds (Gao et al., 2018; Toledano et al., 2021). Zn-coated sulfonated polyetheretherketone has been shown to shift macrophages toward an M2 phenotype while promoting the release of anti-inflammatory and osteogenic cytokines (Liu et al., 2018). These findings reinforce Zn’s status as a “star element” in immunomodulatory scaffold materials (Zamani et al., 2019; Boyd et al., 2009).

Strontium, a non-radioactive element with established osteogenic potential, plays a dual role by inhibiting bone resorption and enhancing bone formation (You et al., 2022). Xu et al. demonstrated that titanium implants with Sr-incorporated micro/nano-roughened surfaces facilitated new bone formation and were associated with increased infiltration of M2 macrophages, along with reduced M1 macrophage presence (Xu et al., 2021).

Copper, in contrast, exerts a predominantly pro-inflammatory effect on macrophages (Flemming, 2023). The rapid ion release from copper oxide nanoparticles has been linked to cytotoxicity and heightened inflammation (Líbalová et al., 2018). Moreover, Cu enhances macrophage signaling by elevating mitochondrial copper levels, an effect associated with CD44 upregulation and immune activation (Solier et al., 2023).

Tantalum, long recognized for its biocompatibility and osteoconductivity (Liu T. et al., 2022; Wang et al., 2022; Woodhall and Spurling, 1945), was once considered biologically inert. However, recent studies reveal that Ta nanoparticles promote M2-like morphology in macrophages, suppress the expression of pro-inflammatory genes, and upregulate anti-inflammatory mediators such as TGF-β1 and IL-10 (Sun et al., 2022).

These findings collectively highlight the capacity of specific metallic elements to shape macrophage polarization and, by extension, modulate the inflammatory milieu in favor of bone regeneration.

However, the immunological roles of metals in scaffold systems remain far from fully elucidated. One critical issue is the dose-dependent duality of several metal ions. For example, while low concentrations of magnesium and zinc are anti-inflammatory, their excessive release may result in cytotoxicity or oxidative stress. Similarly, Copper’s pro-inflammatory effects may be harnessed for early immune activation but could impair healing if not tightly controlled.

Moreover, many current studies fail to account for material interactions, such as the combined effects of metal ions with scaffold stiffness, porosity, or degradation kinetics. The local microenvironment, including pH, ionic strength, and protein adsorption, can significantly alter ion solubility and bioavailability, further complicating interpretation.

Future studies should emphasize systematic dose-response evaluations, longitudinal *in vivo* tracking, and multivariate material design to better understand the dynamic interplay between metal ions, immune responses, and bone remodeling. Such efforts will be essential for developing next-generation immuno-instructive scaffolds that leverage metallic elements with precision and safety.

## 4 Synergistic roles of physical and biochemical cues

The immune microenvironment during bone regeneration is governed by an intricate interplay of mechanical and biochemical signals. While prior sections highlighted how physical cues can guide macrophage polarization, these effects are often modulated or even amplified in the presence of biochemical signals. Increasing evidence suggests that physical and biochemical cues synergize in a spatiotemporally coordinated manner, shaping macrophage phenotypes and downstream regenerative outcomes.

Classic anti-inflammatory cytokines including IL-4, IL-10, and TGF-β have demonstrated efficacy in reducing pathological inflammation both *in vitro* and *in vivo* (Gärtner et al., 2023). IL-4 promotes alternative activation of macrophages toward the M2 phenotype while inhibiting M1 polarization. This shift leads to increased production of IL-10 and TGF-β, further reinforcing the anti-inflammatory microenvironment (Bosurgi et al., 2017). The regulation of these biological agents is a multifaceted and highly interconnected process, with each factor exerting a distinct yet synergistic influence on macrophage behavior and the bone healing cascade.

A representative delivery strategy involves poly (lactic-co-glycolic acid)–multistage porous silicon vector composite microspheres encapsulating IL-4, which upregulate genes associated with M2 polarization such as IL-10, CD206, and Arg1 within 48 h *in vitro* (Minardi et al., 2016). IL-10 inhibits the secretion of proinflammatory cytokines and reactive oxygen species, reducing macrophage activation and infiltration (Shen et al., 2022). TGF-β also suppresses CD4^+^ T cell activation by acting through dendritic cells, promoting the generation of regulatory T cells (Tregs) and increasing IL-10 levels (Orr et al., 2016). Similarly, CXCL12 has been shown to attract M2 macrophages via the CXCR4 receptor axis, and blocking this receptor markedly impairs M2 chemotaxis (Li et al., 2019; Fang et al., 2022).

While many studies have emphasized anti-inflammatory factors, pro-inflammatory mediators like IFN-γ and TNF-α also play critical roles in orchestrating immune responses. IFN-γ promotes M1 polarization and upregulates MHC class II molecule expression, contributing to antigen presentation and early vascularization in bone repair (Liu W. et al., 2022; Luo et al., 2021; Li T. et al., 2018). TNF-α, predominantly produced by macrophages and monocytes (Olszewski et al., 2007), was traditionally viewed as a pro-inflammatory cytokine (Zelová and Hošek, 2013). However, recent evidence suggests that TNF-α possesses dual roles, capable of both amplifying and resolving inflammation depending on concentration, duration, and microenvironmental context (Ma, 2001a). It facilitates leukocyte adhesion via E− and P-selectin expression (Chandrasekharan et al., 2007), but can also suppress IL-12 production (Ma et al., 2000), which in turn may favor M2 polarization due to IL-12’s role as a key proinflammatory mediator (Ma, 2001b; Trinchieri and Gerosa, 1996).

To leverage this immunological complexity, several advanced delivery platforms incorporate both biochemical and physical design elements. For example, Xu et al. engineered genipin-crosslinked carboxymethyl chitosan/collagen hydrogels that acted as a barrier to intercept the initial burst release of IL-4, sustaining a mild M1 response to support mesenchymal stem cell recruitment and angiogenesis in early fracture healing. Over time, degradation of the gel enabled IL-4 release to induce M2 polarization in the remodeling phase (Xu et al., 2023). Similarly, Annamalai et al. created genipin-crosslinked gelatin microspheres loaded with bone morphogenetic protein-2, which were preferentially degraded by M1 macrophages, releasing osteogenic cues in a context-dependent manner (Annamalai et al., 2018).

Recent advances suggest that biomimetic modifications to delivery platforms can substantially improve the immune-instructive potential of biological agents. For example, coating nanoparticles with macrophage or leukocyte-derived membranes enables homotypic targeting and facilitates immune evasion, leading to more efficient delivery and activation of macrophage polarization pathways *in situ* (Savchenko et al., 2023). Such systems mimic natural cell–cell communication, thus providing not only delivery precision but also a biomimetic immune modulation mechanism that may better recapitulate *in vivo* immune signaling. Despite growing evidence supporting the immunomodulatory potential of biological agents, their delivery remains a challenging task, particularly when aiming to achieve synergistic regulation with physical cues. Many current strategies rely on the administration of single cytokines, overlooking the complexity of the *in vivo* microenvironment, which features a dynamic interplay of overlapping and sometimes conflicting signals. In reality, the bioactivity of cytokines is highly dependent on mechanical context, receptor expression patterns, and spatiotemporal interactions with other signaling inputs. As such, single-factor approaches may fail to recapitulate the integrated signaling required for effective macrophage reprogramming and bone repair.

Moreover, the timing of cytokine release often fails to match the immunological phases of bone healing. Anti-inflammatory factors released too early may suppress necessary M1-driven inflammation, while delayed release may miss the critical window for M2 polarization. Similarly, the temporal dynamics of physical cues—such as matrix stiffness—must also align with biological signaling to guide proper immune responses. A mismatch between scaffold degradation, mechanical adaptation, and cytokine kinetics may impair therapeutic outcomes.

Interindividual variability in immune status, vascularization, and comorbid conditions further complicates the predictability and consistency of these immune-material interactions, underscoring the need for adaptable, responsive systems.

Looking ahead, next-generation biomaterials must integrate both spatiotemporal control of biochemical factor release and adaptive physical modulation. Intelligent platforms that respond to environmental triggers, such as pH, enzymatic activity, and ROS, can dynamically tune both cytokine delivery and mechanical properties in real time. When coupled with imaging-guided feedback systems and multi-cytokine delivery logic, these dual-instructive materials may offer a transformative strategy to synchronize immune modulation with the staged requirements of bone regeneration.

## 5 Conclusion

This review highlights the immunoregulatory effects of physical cues in biomaterials for bone tissue engineering, with an emphasis on how these cues influence macrophage polarization and ultimately affect bone regeneration outcomes. Scaffold characteristics serve not only as structural frameworks but also as active regulators of the immune microenvironment.

While earlier scaffold designs prioritized biocompatibility and osteoconduction, it is now increasingly recognized that modulation of the local immune response is equally critical. In particular, macrophages are emerging as key intermediaries between materials and host tissues. Numerous studies have demonstrated the potential of engineered physical cues to shift macrophage behavior toward pro-regenerative phenotypes, especially M2-like phenotypes.

However, despite these promising insights, several challenges remain. Most current studies focus on correlational observations between scaffold cues and macrophage phenotypes, without fully elucidating the underlying mechanosensing mechanisms. While integrin-mediated pathways, such as the integrin–focal adhesion–stress fiber–YAP/TAZ axis, have been proposed, this model may be insufficient to explain macrophage responses in 3D environments. Recent findings suggest that podosomes and filopodia may play dominant roles in macrophage mechanosensation (Cervero et al., 2012), highlighting the need to revisit classic models (Halder et al., 2012; Ohashi et al., 2017).

Moreover, the review primarily focuses on macrophage–scaffold interactions, while neglecting the crosstalk between macrophages and other immune cells, such as T cells and neutrophils, which are increasingly implicated in bone remodeling, inflammation resolution, and osteoclastogenesis (Li J. et al., 2018; Zhang et al., 2021). Future scaffold designs must incorporate this multicellular immune complexity to fully harness the regenerative potential of the host immune system.

To facilitate a holistic understanding, Table 1 summarizes the distinct physical cues, their corresponding effects on macrophage polarization, and the underlying signaling pathways. This integrative comparison highlights the multifactorial and context-dependent nature of scaffold–immune interactions and provides a practical reference for rational design of immuno-instructive biomaterials.

**TABLE 1:** Comparison of physical cues on macrophage modulation.

Physical cue	Effect on macrophage phenotype	Key signaling pathways	References
Stiffness	High stiffness activates Piezo1 leading to Ca^2+^ influx and YAP nuclear translocation, inducing M1 phenotype	Piezo1–YAP, NF-κB	71, 73
	Intermediate stiffness promotes M2 phenotype via ROS reduction and NF-κB inhibition	ROS–NF-κB, Integrin	79
	High stiffness enhances ROCK activity, increases stress fiber formation and inflammation	ROCK, Cytoskeleton tension	84
Viscoelasticity	High viscoelasticity enhances STAT6 activation and cytoskeletal softening, driving M2 polarization	STAT6, Integrin-cytoskeleton	87, 88
	Viscoelastic cues induce metabolic reprogramming from glycolysis to OXPHOS, promoting M2 phenotype	OXPHOS, NF-κB	90
	Modulates LPS signaling duration, enhancing fine-tuned immune responses	LPS–NF-κB	91
Topography	Elongated cell morphology induces M2 marker expression and reduces inflammatory cytokines	Actomyosin, RhoA–ROCK–NF-κB	94, 95
	Nanostructures activate integrin–PI3K–Akt signaling, favoring M2 phenotype	Integrin–PI3K–Akt	101, 102
	75 nm nanospheres reduce early inflammation and promote bone formation *in vivo*	Surface Topography, Osteocalcin	104
Pore Size & Porosity	Small pores induce spatial confinement and promote M1 phenotype	Hypoxia, NF-κB	112, 117
	Large pores enhance oxygen diffusion and cell spreading, inducing M2 phenotype	Oxygenation, Arg1/CD206	111
Hydrophilicity	Hydrophilic surfaces modulate protein adsorption and upregulate IL-10 to promote M2 polarization	Protein corona, IL-10/IL-4	119, 120
	Hydrophilic titanium surfaces enhance osseointegration and anti-inflammatory cytokine expression	Anti-inflammatory cytokines	120
Electromagnetic Stimuli	Ultrasound-stimulated piezoelectric materials induce Ca^2+^ signaling and M2 polarization	Ca^2+^–CAMK2A–NF-κB, OXPHOS	125, 128
	Square waves enhance M1; sinusoidal waves modulate both M1 and M2 polarization	Waveform–dependent modulation	129, 130
Metal Composition	Mg, Zn, Sr, Ta promote M2 via NF-κB inhibition and upregulation of IL-10 and PPARγ	NF-κB inhibition, PPARγ, IL-10	132, 138, 142
	Cu promotes M1 phenotype via mitochondrial copper signaling and CD44 upregulation	Mitochondrial Cu, CD44, ROS	143, 145

Additionally, the cytokine requirements during bone regeneration are highly dynamic, varying across the inflammatory, repair, and remodeling phases. Coupled with the fact that scaffold degradation continuously reshapes the microenvironment sensed by macrophages (de Jonge et al., 2014; Wissing et al., 2019), this necessitates the development of materials capable of spatiotemporally controlled immunomodulation (Table 2).

**TABLE 2:** Future challenges and design guidelines for immuno-instructive biomaterials.

Challenges	Future directions	Design guidelines
Limited understanding of macrophage mechanosensing mechanisms	Elucidate signaling pathways involving podosomes and filopodia	Incorporate roles of non-canonical mechanosensory structures like podosomes
Insufficient models reflecting 3D physiological environments	Develop more physiologically relevant 3D mechanotransduction models	Mimic 3D microenvironments in scaffold design
Neglect of immune crosstalk with T/B cells and other immune players	Investigate interactions between macrophages and T/B cells in bone remodeling	Integrate multicellular immune interactions in scaffold design
Dynamic cytokine demands during regeneration phases	Construct materials enabling phase-specific immune modulation	Enable stage-specific release of immunoregulatory signals
Scaffold degradation alters local microenvironment over time	Develop spatiotemporally responsive materials for sustained immunomodulation	Design biodegradable scaffolds responsive to environmental and temporal cues

In conclusion, this review presents a novel conceptual framework in which biomaterial physical cues are positioned as central regulators of immune response in bone regeneration. Moving forward, integrating physical, chemical, and temporal dimensions of macrophage modulation—alongside mechanistic insights and multicellular immune integration—will be crucial to designing immuno-instructive biomaterials for robust, functional bone repair.

## References

[B1] Aderem A. Underhill D. M. (1999). Mechanisms of phagocytosis in macrophages. Annu. Rev. Immunol. 17 (1), 593–623. 10.1146/annurev.immunol.17.1.593 10358769

[B2] Alexander K. A. Chang M. K. Maylin E. R. Kohler T. Müller R. Wu A. C. (2011). Osteal macrophages promote *in vivo* intramembranous bone healing in a mouse tibial injury model. J. Bone Mineral Res. 26 (7), 1517–1532. 10.1002/jbmr.354 21305607

[B3] Annamalai R. T. Turner P. A. Carson W. F. Levi B. Kunkel S. Stegemann J. P. (2018). Harnessing macrophage-mediated degradation of gelatin microspheres for spatiotemporal control of BMP2 release. Biomaterials 161, 216–227. 10.1016/j.biomaterials.2018.01.040 29421557 PMC5831261

[B4] Argyle D. Kitamura T. (2018). Targeting macrophage-recruiting chemokines as a novel therapeutic strategy to prevent the progression of solid tumors. Front. Immunol. 9, 2629. 10.3389/fimmu.2018.02629 30483271 PMC6243037

[B5] Ariganello M. B. Guadarrama Bello D. Rodriguez-Contreras A. Sadeghi S. Isola G. Variola F. (2018). Surface nanocavitation of titanium modulates macrophage activity. Int. J. Nanomedicine 13, 8297–8308. 10.2147/IJN.S185436 30584301 PMC6287524

[B6] Atcha H. Jairaman A. Holt J. R. Meli V. S. Nagalla R. R. Veerasubramanian P. K. (2021). Mechanically activated ion channel Piezo1 modulates macrophage polarization and stiffness sensing. Nat. Commun. 12 (1), 3256. 10.1038/s41467-021-23482-5 34059671 PMC8167181

[B7] Batoon L. Millard S. M. Wullschleger M. E. Preda C. Wu A. C. K. Kaur S. (2019). CD169+ macrophages are critical for osteoblast maintenance and promote intramembranous and endochondral ossification during bone repair. Biomaterials 196, 51–66. 10.1016/j.biomaterials.2017.10.033 29107337

[B8] Bessa-Gonçalves M. Ribeiro-Machado C. Costa M. Ribeiro C. Barbosa J. Barbosa M. (2023). Magnesium incorporation in fibrinogen scaffolds promotes macrophage polarization towards M2 phenotype. Acta Biomater. 155, 667–683. 10.1016/j.actbio.2022.10.046 36328124

[B9] Blakney A. K. Swartzlander M. D. Bryant S. J. (2012). The effects of substrate stiffness on the *in vitro* activation of macrophages and *in vivo* host response to poly(ethylene glycol)-based hydrogels. J. Biomed. Mater Res. A 100 (6), 1375–1386. 10.1002/jbm.a.34104 22407522 PMC3339197

[B10] Bosco M. C. (2019). Macrophage polarization: reaching across the aisle? J. Allergy Clin. Immunol. 143 (4), 1348–1350. 10.1016/j.jaci.2018.12.995 30639344

[B11] Bosurgi L. Cao Y. G. Cabeza-Cabrerizo M. Tucci A. Hughes L. D. Kong Y. (2017). Macrophage function in tissue repair and remodeling requires IL-4 or IL-13 with apoptotic cells. Science 356 (6342), 1072–1076. 10.1126/science.aai8132 28495875 PMC5556699

[B12] Bouchery T. Harris N. (2019). Neutrophil-macrophage cooperation and its impact on tissue repair. Immunol. Cell Biol. 97 (3), 289–298. 10.1111/imcb.12241 30710448

[B13] Boyd D. Carroll G. Towler M. R. Freeman C. Farthing P. Brook I. M. (2009). Preliminary investigation of novel bone graft substitutes based on strontium-calcium-zinc-silicate glasses. J. Mater Sci. Mater Med. 20 (1), 413–420. 10.1007/s10856-008-3569-0 18839286

[B14] Burnett S. H. Kershen E. J. Zhang J. Zeng L. Straley S. C. Kaplan A. M. (2004). Conditional macrophage ablation in transgenic mice expressing a Fas-based suicide gene. J. Leukoc. Biol. 75 (4), 612–623. 10.1189/jlb.0903442 14726498

[B15] Carrara S. C. Davila-Lezama A. Cabriel C. Berenschot E. J. Krol S. Gardeniers J. (2023). 3D topographies promote macrophage M2d-Subset differentiation. Mater Today Bio 24, 100897. 10.1016/j.mtbio.2023.100897 PMC10758855 38169974

[B16] Cervero P. Himmel M. Krüger M. Linder S. (2012). Proteomic analysis of podosome fractions from macrophages reveals similarities to spreading initiation centres. Eur. J. Cell Biol. 91 (11-12), 908–922. 10.1016/j.ejcb.2012.05.005 22721921

[B17] Chandrasekharan U. M. Siemionow M. Unsal M. Yang L. Poptic E. Bohn J. (2007). Tumor necrosis factor α (TNF-α) receptor-II is required for TNF-α–induced leukocyte-endothelial interaction *in vivo* . Blood 109 (5), 1938–1944. 10.1182/blood-2006-05-020875 17068152 PMC1801063

[B18] Chang M. K. Raggatt L. J. Alexander K. A. Kuliwaba J. S. Fazzalari N. L. Schroder K. (2008). Osteal tissue macrophages are intercalated throughout human and mouse bone lining tissues and regulate osteoblast function *in vitro* and *in vivo* . J. Immunol. 181 (2), 1232–1244. 10.4049/jimmunol.181.2.1232 18606677

[B19] Chaudhuri O. Cooper-White J. Janmey P. A. Mooney D. J. Shenoy V. B. (2020). Effects of extracellular matrix viscoelasticity on cellular behaviour. Nature 584 (7822), 535–546. 10.1038/s41586-020-2612-2 32848221 PMC7676152

[B20] Chaudhuri O. Gu L. Klumpers D. Darnell M. Bencherif S. A. Weaver J. C. (2016). Hydrogels with tunable stress relaxation regulate stem cell fate and activity. Nat. Mater 15 (3), 326–334. 10.1038/nmat4489 26618884 PMC4767627

[B21] Chen B. Luo L. Wei X. Gong D. Li Z. Li S. (2021). M1 bone marrow-derived macrophage-derived extracellular vesicles inhibit angiogenesis and myocardial regeneration following myocardial infarction *via* the MALAT1/MicroRNA-25-3p/CDC42 axis. Oxid. Med. Cell Longev. 2021, 9959746. 10.1155/2021/9959746 34745428 PMC8570847

[B22] Chen M. Zhang Y. Zhou P. Liu X. Zhao H. Zhou X. (2020). Substrate stiffness modulates bone marrow-derived macrophage polarization through NF-κB signaling pathway. Bioact. Mater 5 (4), 880–890. 10.1016/j.bioactmat.2020.05.004 32637751 PMC7332470

[B23] Chen Z. Klein T. Murray R. Z. Crawford R. Chang J. Wu C. (2016). Osteoimmunomodulation for the development of advanced bone biomaterials. Mater. Today 19 (6), 304–321. 10.1016/j.mattod.2015.11.004

[B24] Cheng H. Bai J. Zhou X. Chen N. Jiang Q. Ren Z. (2024). Electrical stimulation with polypyrrole-coated polycaprolactone/silk fibroin scaffold promotes sacral nerve regeneration by modulating macrophage polarisation. Biomater. Transl. 5 (2), 157–174. 10.12336/biomatertransl.2024.02.006 39351163 PMC11438605

[B25] Cheng P. Weng Z. Hamushan M. Cai W. Zhang Y. Ren Z. (2022). High-purity magnesium screws modulate macrophage polarization during the tendon-bone healing process in the anterior cruciate ligament reconstruction rabbit model. Regen. Biomater. 9, rbac067. 10.1093/rb/rbac067 36284747 PMC9580517

[B26] Chinetti-Gbaguidi G. Baron M. Bouhlel M. A. Vanhoutte J. Copin C. Sebti Y. (2011). Human atherosclerotic plaque alternative macrophages display low cholesterol handling but high phagocytosis because of distinct activities of the PPARγ and LXRα pathways. Circulation Res. 108 (8), 985–995. 10.1161/CIRCRESAHA.110.233775 21350215 PMC3319502

[B27] Cockerill I. Su Y. Sinha S. Qin Y. X. Zheng Y. Young M. L. (2020). Porous zinc scaffolds for bone tissue engineering applications: a novel additive manufacturing and casting approach. Mater Sci. Eng. C Mater Biol. Appl. 110, 110738. 10.1016/j.msec.2020.110738 32204047 PMC7096330

[B28] Cui Y. Li X. He X. Zhou X. Wang X. Lin K. (2025). Schwann cell-derived exosomes accelerate periodontal bone regeneration with osteogenesis, angiogenesis, and neurogenesis. J. Mater Chem. B 13 (12), 4020–4029. 10.1039/d4tb02601b 40040598

[B29] Dare A. J. Hu G. (2017). China’s evolving fracture burden. Lancet Glob. Health 5 (8), e736–e737. 10.1016/S2214-109X(17)30254-1 28666815

[B30] de Jonge N. Foolen J. Brugmans M. C. P. Söntjens S. H. M. Baaijens F. P. T. Bouten C. V. C. (2014). Degree of scaffold degradation influences collagen (re)orientation in engineered tissues. Tissue Eng. Part A 20 (11-12), 1747–1757. 10.1089/ten.TEA.2013.0517 24372199

[B31] de Oliveira S. Rosowski E. E. Huttenlocher A. (2016). Neutrophil migration in infection and wound repair: going forward in reverse. Nat. Rev. Immunol. 16 (6), 378–391. 10.1038/nri.2016.49 27231052 PMC5367630

[B32] Díaz-Bulnes P. Saiz M. L. López-Larrea C. Rodríguez R. M. (2020). Crosstalk between hypoxia and ER stress response: a key regulator of macrophage polarization. Front. Immunol. 10, 2951. 10.3389/fimmu.2019.02951 31998288 PMC6961549

[B33] Dinnes D. L. M. Marçal H. Mahler S. M. Santerre J. P. Labow R. S. (2007). Material surfaces affect the protein expression patterns of human macrophages: a proteomics approach. J. Biomed. Mater Res. A 80 (4), 895–908. 10.1002/jbm.a.30967 17072854

[B34] Dos Anjos Cassado A. (2017). F4/80 as a major macrophage marker: the case of the peritoneum and spleen. Results Probl. Cell Differ. 62, 161–179. 10.1007/978-3-319-54090-0_7 28455709

[B35] Duan J. X. Zhou Y. Zhou A. Y. Guan X. X. Liu T. Yang H. H. (2017). Calcitonin gene-related peptide exerts anti-inflammatory property through regulating murine macrophages polarization *in vitro* . Mol. Immunol. 91, 105–113. 10.1016/j.molimm.2017.08.020 28892747

[B36] Ehterami A. Kazemi M. Nazari B. Saraeian P. Azami M. (2018). Fabrication and characterization of highly porous barium titanate based scaffold coated by Gel/HA nanocomposite with high piezoelectric coefficient for bone tissue engineering applications. J. Mech. Behav. Biomed. Mater. 79, 195–202. 10.1016/j.jmbbm.2017.12.034 29306083

[B37] Elefteriou F. (2018). Impact of the autonomic nervous system on the skeleton. Physiol. Rev. 98 (3), 1083–1112. 10.1152/physrev.00014.2017 29717928 PMC6088147

[B38] Fang J. Y. Yang Z. Hu W. Hoang B. X. Han B. (2025). Viscoelastic hydrogel modulates phenotype of macrophage-derived multinucleated cells and macrophage differentiation in foreign body reactions. J. Biomed. Mater Res. A 113 (1), e37814. 10.1002/jbm.a.37814 39429027

[B39] Fang X. Y. Zhan Y. X. Zhou X. M. Wu L. Lin J. Yi Y. (2022). CXCL12/CXCR4 mediates orthodontic root resorption *via* regulating the M1/M2 ratio. J. Dent. Res. 101 (5), 569–579. 10.1177/00220345211050324 34847760

[B40] Flemming A. (2023). Copper boosts pro-inflammatory state of macrophages. Nat. Rev. Immunol. 23 (6), 344. 10.1038/s41577-023-00889-3 PMC10176277 37173540

[B41] Folkman J. Moscona A. (1978). Role of cell shape in growth control. Nature 273 (5661), 345–349. 10.1038/273345a0 661946

[B42] Frith J. C. Mönkkönen J. Blackburn G. M. Russell R. G. Rogers M. J. (1997). Clodronate and liposome-encapsulated clodronate are metabolized to a toxic ATP analog, adenosine 5’-(beta, gamma-dichloromethylene) triphosphate, by Mammalian cells *in vitro* . J. Bone Min. Res. 12 (9), 1358–1367. 10.1359/jbmr.1997.12.9.1358 9286751

[B43] Gao H. Dai W. Zhao L. Min J. Wang F. (2018). The role of zinc and zinc homeostasis in macrophage function. J. Immunol. Res. 2018, 6872621–11. 10.1155/2018/6872621 30622979 PMC6304900

[B44] Gärtner Y. Bitar L. Zipp F. Vogelaar C. F. (2023). Interleukin-4 as a therapeutic target. Pharmacol. Ther. 242, 108348. 10.1016/j.pharmthera.2023.108348 36657567

[B45] GBD 2019 Diseases and Injuries Collaborators, Vos T. Lim S. S. Abbafati C. Abbas K. M. Abbasi M. Abbasifard M. (2020). Global burden of 369 diseases and injuries in 204 countries and territories, 1990-2019: a systematic analysis for the global burden of disease study 2019. Lancet 396 (10258), 1204–1222. 10.1016/S0140-6736(20)30925-9 33069326 PMC7567026

[B46] Gordon S. Hamann J. Lin H. H. Stacey M. (2011). F4/80 and the related adhesion-GPCRs. Eur. J. Immunol. 41 (9), 2472–2476. 10.1002/eji.201141715 21952799

[B47] Gou M. Wang H. Xie H. Song H. (2024). Macrophages in guided bone regeneration: potential roles and future directions. Front. Immunol. 15, 1396759. 10.3389/fimmu.2024.1396759 38736888 PMC11082316

[B48] Gu J. He X. Chen X. Dong L. Weng W. Cheng K. (2022). Effects of electrical stimulation on cytokine-induced macrophage polarization. J. Tissue Eng. Regen. Med. 16 (5), 448–459. 10.1002/term.3292 35225425

[B49] Guihard P. Boutet M. A. Brounais-Le Royer B. Gamblin A. L. Amiaud J. Renaud A. (2015). Oncostatin M, an inflammatory cytokine produced by macrophages, supports intramembranous bone healing in a mouse model of tibia injury. Am. J. Pathology 185 (3), 765–775. 10.1016/j.ajpath.2014.11.008 25559270

[B50] Guo D. Lin C. Lu Y. Guan H. Qi W. Zhang H. (2022). FABP4 secreted by M1-polarized macrophages promotes synovitis and angiogenesis to exacerbate rheumatoid arthritis. Bone Res. 10, 45. 10.1038/s41413-022-00211-2 35729106 PMC9213409

[B51] Halder G. Dupont S. Piccolo S. (2012). Transduction of mechanical and cytoskeletal cues by YAP and TAZ. Nat. Rev. Mol. Cell Biol. 13 (9), 591–600. 10.1038/nrm3416 22895435

[B52] Hamlet S. M. Lee R. S. B. Moon H. J. Alfarsi M. A. Ivanovski S. (2019). Hydrophilic titanium surface-induced macrophage modulation promotes pro-osteogenic signalling. Clin. Oral Implants Res. 30 (11), 1085–1096. 10.1111/clr.13522 31397920

[B53] Hoare J. I. Rajnicek A. M. McCaig C. D. Barker R. N. Wilson H. M. (2016). Electric fields are novel determinants of human macrophage functions. J. Leukoc. Biol. 99 (6), 1141–1151. 10.1189/jlb.3A0815-390R 26718542

[B54] Hotchkiss K. M. Reddy G. B. Hyzy S. L. Schwartz Z. Boyan B. D. Olivares-Navarrete R. (2016). Titanium surface characteristics, including topography and wettability, alter macrophage activation. Acta Biomater. 31, 425–434. 10.1016/j.actbio.2015.12.003 26675126 PMC4728000

[B55] Hu T. Xu H. Wang C. Qin H. An Z. (2018). Magnesium enhances the chondrogenic differentiation of mesenchymal stem cells by inhibiting activated macrophage-induced inflammation. Sci. Rep. 8 (1), 3406. 10.1038/s41598-018-21783-2 29467509 PMC5821731

[B56] Hu W. Deng J. Su Z. Wang H. Lin S. (2024). Advances on T cell immunity in bone remodeling and bone regeneration. Zhejiang Da Xue Xue Bao Yi Xue Ban. 53 (4), 450–459. 10.3724/zdxbyxb-2023-0619 39183057 PMC11375490

[B57] Huang J. H. He H. Chen Y. N. Liu Z. Romani M. D. Xu Z. Y. (2022). Exosomes derived from M2 macrophages improve angiogenesis and functional recovery after spinal cord injury through HIF-1α/VEGF axis. Brain Sci. 12 (10), 1322. 10.3390/brainsci12101322 36291255 PMC9599527

[B58] Huang X. Li Y. Fu M. Xin H. B. (2018). Polarizing macrophages *in vitro* . Methods Mol. Biol. 1784, 119–126. 10.1007/978-1-4939-7837-3_12 29761394 PMC8875934

[B59] Hume D. A. Loutit J. F. Gordon S. (1984). The mononuclear phagocyte system of the mouse defined by immunohistochemical localization of antigen f4/80: macrophages of bone and associated connective tissue. J. Cell Sci. 66 (1), 189–194. 10.1242/jcs.66.1.189 6378941

[B60] Irwin E. F. Saha K. Rosenbluth M. Gamble L. J. Castner D. G. Healy K. E. (2008). Modulus-dependent macrophage adhesion and behavior. J. Biomaterials Sci. Polym. Ed. 19 (10), 1363–1382. 10.1163/156856208786052407 18854128

[B61] Jetten N. Verbruggen S. Gijbels M. J. Post M. J. De Winther M. P. J. Donners MMPC (2014). Anti-inflammatory M2, but not pro-inflammatory M1 macrophages promote angiogenesis *in vivo* . Angiogenesis 17 (1), 109–118. 10.1007/s10456-013-9381-6 24013945

[B62] Jiang S. Lyu C. Zhao P. Li W. Kong W. Huang C. (2019). Cryoprotectant enables structural control of porous scaffolds for exploration of cellular mechano-responsiveness in 3D. Nat. Commun. 10 (1), 3491. 10.1038/s41467-019-11397-1 31375674 PMC6677882

[B63] Jin S. S. He D. Q. Luo D. Wang Y. Yu M. Guan B. (2019). A biomimetic hierarchical nanointerface orchestrates macrophage polarization and mesenchymal stem cell recruitment to promote endogenous bone regeneration. ACS Nano 13 (6), 6581–6595. 10.1021/acsnano.9b00489 31125522

[B64] Kalashnikov N. Moraes C. (2023). Substrate viscoelasticity affects human macrophage morphology and phagocytosis. Soft Matter 19 (13), 2438–2445. 10.1039/d2sm01683d 36930245

[B65] Karazisis D. Petronis S. Agheli H. Emanuelsson L. Norlindh B. Johansson A. (2017). The influence of controlled surface nanotopography on the early biological events of osseointegration. Acta Biomater. 53, 559–571. 10.1016/j.actbio.2017.02.026 28232253

[B66] Kim H. Wang S. Y. Kwak G. Yang Y. Kwon I. C. Kim S. H. (2019). Exosome-guided phenotypic switch of M1 to M2 macrophages for cutaneous wound healing. Adv. Sci. (Weinh) 6 (20), 1900513. 10.1002/advs.201900513 31637157 PMC6794619

[B67] Kong Y. Liu F. Ma B. Duan J. Yuan W. Sang Y. (2021). Wireless localized electrical stimulation generated by an ultrasound‐driven piezoelectric discharge regulates proinflammatory macrophage polarization. Adv. Sci. (Weinh) 8 (13), 2100962. 10.1002/advs.202100962 34258169 PMC8261497

[B68] Kuboki Y. Jin Q. Kikuchi M. Mamood J. Takita H. (2002). Geometry of artificial ECM: sizes of pores controlling phenotype expression in BMP-Induced osteogenesis and chondrogenesis. Connect. Tissue Res. 43 (2-3), 529–534. 10.1080/03008200290001104 12489210

[B69] Labernadie A. Thibault C. Vieu C. Maridonneau-Parini I. Charrière G. M. (2010). Dynamics of podosome stiffness revealed by atomic force microscopy. Proc. Natl. Acad. Sci. 107 (49), 21016–21021. 10.1073/pnas.1007835107 21081699 PMC3000246

[B70] Lai Y. Wahyuningtyas R. Aui S. Chang K. (2019). Autocrine VEGF signalling on M2 macrophages regulates PD‐L1 expression for immunomodulation of T cells. J. Cell Mol. Med. 23 (2), 1257–1267. 10.1111/jcmm.14027 30456891 PMC6349155

[B71] Lee M. Du H. Winer D. A. Clemente-Casares X. Tsai S. (2022). Mechanosensing in macrophages and dendritic cells in steady-state and disease. Front. Cell Dev. Biol. 10, 1044729. 10.3389/fcell.2022.1044729 36467420 PMC9712790

[B72] Li J. Tan J. Martino M. M. Lui K. O. (2018b). Regulatory T-Cells: potential regulator of tissue repair and regeneration. Front. Immunol. 9, 585. 10.3389/fimmu.2018.00585 29662491 PMC5890151

[B73] Li T. Peng M. Yang Z. Zhou X. Deng Y. Jiang C. (2018a). 3D-printed IFN-γ-loading calcium silicate-β-tricalcium phosphate scaffold sequentially activates M1 and M2 polarization of macrophages to promote vascularization of tissue engineering bone. Acta Biomater. 71, 96–107. 10.1016/j.actbio.2018.03.012 29549051

[B74] Li X. Bu W. Meng L. Liu X. Wang S. Jiang L. (2019). CXCL12/CXCR4 pathway orchestrates CSC-Like properties by CAF recruited tumor associated macrophage in OSCC. Exp. Cell Res. 378 (2), 131–138. 10.1016/j.yexcr.2019.03.013 30857971

[B75] Li Z. Bratlie K. M. (2021). Effect of RGD functionalization and stiffness of gellan gum hydrogels on macrophage polarization and function. Mater. Sci. Eng. C 128, 112303. 10.1016/j.msec.2021.112303 34474854

[B76] Líbalová H. Costa P. M. Olsson M. Farcal L. Ortelli S. Blosi M. (2018). Toxicity of surface-modified copper oxide nanoparticles in a mouse macrophage cell line: interplay of particles, surface coating and particle dissolution. Chemosphere 196, 482–493. 10.1016/j.chemosphere.2017.12.182 29324388

[B77] Lim J. E. Chung E. Son Y. (2017). A neuropeptide, Substance-P, directly induces tissue-repairing M2 like macrophages by activating the PI3K/Akt/mTOR pathway Even in the presence of IFNγ. Sci. Rep. 7 (1), 9417. 10.1038/s41598-017-09639-7 28842601 PMC5573373

[B78] Lin R. Ge K. Fan D. Li J. Zhou G. Zhang K. (2023). Multi-walled carbon nanotubes reversing the bone formation of bone marrow stromal cells by activating M2 macrophage polarization. Regen. Biomater. 10, rbad042. 10.1093/rb/rbad042 37274617 PMC10234760

[B79] Linder S. Wiesner C. (2016). Feel the force: podosomes in mechanosensing. Exp. Cell Res. 343 (1), 67–72. 10.1016/j.yexcr.2015.11.026 26658516

[B80] Liu L. Huang T. Xie Z. Ye Z. Zhang J. Liao H. (2023a). Liquid crystalline matrix-induced viscoelastic mechanical stimulation modulates activation and phenotypes of macrophage. J. Biomater. Appl. 37 (9), 1568–1581. 10.1177/08853282221136580 36917676

[B81] Liu S. Chen J. Shi J. Zhou W. Wang L. Fang W. (2020). M1-like macrophage-derived exosomes suppress angiogenesis and exacerbate cardiac dysfunction in a myocardial infarction microenvironment. Basic Res. Cardiol. 115 (2), 22. 10.1007/s00395-020-0781-7 32112145

[B82] Liu T. Li B. Chen G. Ye X. Zhang Y. (2022a). Nano tantalum-coated 3D printed porous polylactic acid/beta-tricalcium phosphate scaffolds with enhanced biological properties for guided bone regeneration. Int. J. Biol. Macromol. 221, 371–380. 10.1016/j.ijbiomac.2022.09.003 36067849

[B83] Liu W. Li J. Cheng M. Wang Q. Yeung K. W. K. Chu P. K. (2018). Zinc-modified sulfonated polyetheretherketone surface with immunomodulatory function for guiding cell fate and bone regeneration. Adv. Sci. (Weinh) 5 (10), 1800749. 10.1002/advs.201800749 30356934 PMC6193167

[B84] Liu W. Zhang S. Wang J. (2022b). IFN-γ, should not be ignored in SLE. Front. Immunol. 13, 954706. 10.3389/fimmu.2022.954706 36032079 PMC9399831

[B85] Liu Y. Suarez-Arnedo A. Riley L. Miley T. Xia J. Segura T. (2023b). Spatial confinement modulates macrophage response in microporous annealed particle (MAP) scaffolds. Adv. Healthc. Mater. 12 (26), 2300823. 10.1002/adhm.202300823 PMC10592513 37165945

[B86] Locati M. Curtale G. Mantovani A. (2020). Diversity, mechanisms, and significance of macrophage plasticity. Annu. Rev. Pathol. Mech. Dis. 15 (1), 123–147. 10.1146/annurev-pathmechdis-012418-012718 PMC7176483 31530089

[B87] Loi F. Córdova L. A. Zhang R. Pajarinen J. Lin T. h. Goodman S. B. (2016). The effects of immunomodulation by macrophage subsets on osteogenesis *in vitro* . Stem Cell Res. and Ther. 7 (1), 15. 10.1186/s13287-016-0276-5 26801095 PMC4724110

[B88] Luo M. Zhao F. Liu L. Yang Z. Tian T. Chen X. (2021). IFN-γ/SrBG composite scaffolds promote osteogenesis by sequential regulation of macrophages from M1 to M2. J. Mater Chem. B 9 (7), 1867–1876. 10.1039/d0tb02333g 33533360

[B89] Lurier E. B. Dalton D. Dampier W. Raman P. Nassiri S. Ferraro N. M. (2017). Transcriptome analysis of IL-10-stimulated (M2c) macrophages by next-generation sequencing. Immunobiology 222 (7), 847–856. 10.1016/j.imbio.2017.02.006 28318799 PMC5719494

[B90] Ma X. (2001a). TNF-Alpha and IL-12: a balancing act in macrophage functioning. Microbes Infect. 3 (2), 121–129. 10.1016/s1286-4579(00)01359-9 11251298

[B91] Ma X. (2001b). TNF-α and IL-12:a balancing act in macrophage functioning. Microbes Infect. 3 (2), 121–129. 10.1016/S1286-4579(00)01359-9 11251298

[B92] Ma X. Sun J. Papasavvas E. Riemann H. Robertson S. Marshall J. (2000). Inhibition of IL-12 production in human monocyte-derived macrophages by TNF. J. Immunol. 164 (4), 1722–1729. 10.4049/jimmunol.164.4.1722 10657616

[B93] Madden L. R. Mortisen D. J. Sussman E. M. Dupras S. K. Fugate J. A. Cuy J. L. (2010). Proangiogenic scaffolds as functional templates for cardiac tissue engineering. Proc. Natl. Acad. Sci. U. S. A. 107 (34), 15211–15216. 10.1073/pnas.1006442107 20696917 PMC2930533

[B94] Maksimkin A. V. Senatov F. S. Anisimova N. Y. Kiselevskiy M. Zalepugin D. Chernyshova I. (2017). Multilayer porous UHMWPE scaffolds for bone defects replacement. Mater Sci. Eng. C Mater Biol. Appl. 73, 366–372. 10.1016/j.msec.2016.12.104 28183620

[B95] Mann E. R. Li X. (2014). Intestinal antigen-presenting cells in mucosal immune homeostasis: crosstalk between dendritic cells, macrophages and B-cells. World J. Gastroenterol. 20 (29), 9653–9664. 10.3748/wjg.v20.i29.9653 25110405 PMC4123356

[B96] McCauley J. Bitsaktsis C. Cottrell J. (2020). Macrophage subtype and cytokine expression characterization during the acute inflammatory phase of mouse bone fracture repair. J. Orthop. Res. 38 (8), 1693–1702. 10.1002/jor.24603 31989683

[B97] McWhorter F. Y. Wang T. Nguyen P. Chung T. Liu W. F. (2013). Modulation of macrophage phenotype by cell shape. Proc. Natl. Acad. Sci. U. S. A. 110 (43), 17253–17258. 10.1073/pnas.1308887110 24101477 PMC3808615

[B98] Mehdizadeh H. Sumo S. Bayrak E. S. Brey E. M. Cinar A. (2013). Three-dimensional modeling of angiogenesis in porous biomaterial scaffolds. Biomaterials 34 (12), 2875–2887. 10.1016/j.biomaterials.2012.12.047 23357368

[B99] Mei F. Guo Y. Wang Y. Zhou Y. Heng B. C. Xie M. (2024). Matrix stiffness regulates macrophage polarisation *via* the Piezo1‐YAP signalling axis. Cell Prolif. 57 (8), e13640. 10.1111/cpr.13640 38556840 PMC11294424

[B100] Meli V. S. Atcha H. Veerasubramanian P. K. Nagalla R. R. Luu T. U. Chen E. Y. (2020). YAP-Mediated mechanotransduction tunes the macrophage inflammatory response. Sci. Adv. 6 (49), eabb8471. 10.1126/sciadv.abb8471 33277245 PMC7717914

[B101] Michiels R. Gensch N. Erhard B. Rohrbach A. (2022). Pulling, failing, and adaptive mechanotransduction of macrophage filopodia. Biophys. J. 121 (17), 3224–3241. 10.1016/j.bpj.2022.07.028 35927956 PMC9463700

[B102] Minardi S. Corradetti B. Taraballi F. Byun J. H. Cabrera F. Liu X. (2016). IL-4 release from a biomimetic scaffold for the temporally controlled modulation of macrophage response. Ann. Biomed. Eng. 44 (6), 2008–2019. 10.1007/s10439-016-1580-z 26951461

[B103] Muangsanit P. Yuddnaraveesak P. Singhatanadgit W. (2025). Regenerative potential of neural stem/progenitor cells for bone repair. Tissue Eng. Part B Rev. 10.1089/ten.teb.2024.0188 39761109

[B104] Nobs S. P. Kopf M. (2021). Tissue-resident macrophages: guardians of organ homeostasis. Trends Immunol. 42 (6), 495–507. 10.1016/j.it.2021.04.007 33972166

[B105] Ohashi K. Fujiwara S. Mizuno K. (2017). Roles of the cytoskeleton, cell adhesion and rho signalling in mechanosensing and mechanotransduction. J. Biochem. 161 (3), 245–254. 10.1093/jb/mvw082 28082721

[B106] Olszewski M. B. Groot A. J. Dastych J. Knol E. F. (2007). TNF trafficking to human mast cell granules: mature chain-dependent endocytosis. J. Immunol. 178 (9), 5701–5709. 10.4049/jimmunol.178.9.5701 17442953

[B107] Orr S. Strominger I. Eremenko E. Vinogradov E. Ruvinov E. Monsonego A. (2016). TGF-β affinity-bound to a macroporous alginate scaffold generates local and peripheral immunotolerant responses and improves allocell transplantation. Acta Biomater. 45, 196–209. 10.1016/j.actbio.2016.08.015 27523029

[B108] Ovchinnikov D. A. (2008). Macrophages in the embryo and beyond: much more than just giant phagocytes. genesis 46 (9), 447–462. 10.1002/dvg.20417 18781633

[B109] Pajarinen J. Lin T. Gibon E. Kohno Y. Maruyama M. Nathan K. (2019). Mesenchymal stem cell-macrophage crosstalk and bone healing. Biomaterials 196, 80–89. 10.1016/j.biomaterials.2017.12.025 29329642 PMC6028312

[B110] Perdiguero E. G. Geissmann F. (2016). The development and maintenance of resident macrophages. Nat. Immunol. 17 (1), 2–8. 10.1038/ni.3341 26681456 PMC4950995

[B111] Pujari-Palmer S. Chen S. Rubino S. Weng H. Xia W. Engqvist H. (2016). *In vivo* and *in vitro* evaluation of hydroxyapatite nanoparticle morphology on the acute inflammatory response. Biomaterials 90, 1–11. 10.1016/j.biomaterials.2016.02.039 26974703

[B112] Qiao X. Wang H. He Y. Song D. Altawil A. Wang Q. (2023). Grape seed proanthocyanidin ameliorates LPS-Induced acute lung injury by modulating M2a macrophage polarization *via* the TREM2/PI3K/Akt pathway. Inflammation 46 (6), 2147–2164. 10.1007/s10753-023-01868-5 37566293 PMC10673742

[B113] Raggatt L. J. Wullschleger M. E. Alexander K. A. Wu A. C. Millard S. M. Kaur S. (2014). Fracture healing *via* periosteal callus formation requires macrophages for both initiation and progression of early endochondral ossification. Am. J. Pathology 184 (12), 3192–3204. 10.1016/j.ajpath.2014.08.017 25285719

[B114] Rooijen N. van Kesteren-Hendrikx E. van (2003). *In vivo* Depletion of Macrophages by Liposome-Mediated Suicide, Methods Enzym. 373, 3–16. 10.1016/S0076-6879(03)73001-8 14714393

[B115] Saino E. Focarete M. L. Gualandi C. Emanuele E. Cornaglia A. I. Imbriani M. (2011). Effect of electrospun fiber diameter and alignment on macrophage activation and secretion of proinflammatory cytokines and chemokines. Biomacromolecules 12 (5), 1900–1911. 10.1021/bm200248h 21417396

[B116] Savchenko I. V. Zlotnikov I. D. Kudryashova E. V. (2023). Biomimetic systems involving macrophages and their potential for targeted drug delivery. Biomimetics 8 (7), 543. 10.3390/biomimetics8070543 37999184 PMC10669405

[B117] Schall T. J. Proudfoot A. E. I. (2011). Overcoming hurdles in developing successful drugs targeting chemokine receptors. Nat. Rev. Immunol. 11 (5), 355–363. 10.1038/nri2972 21494268

[B118] Schlundt C. El Khassawna T. Serra A. Dienelt A. Wendler S. Schell H. (2018). Macrophages in bone fracture healing: their essential role in endochondral ossification. Bone 106, 78–89. 10.1016/j.bone.2015.10.019 26529389

[B119] Schmidt-Bleek K. Petersen A. Dienelt A. Schwarz C. Duda G. N. (2014). Initiation and early control of tissue regeneration – bone healing as a model system for tissue regeneration. Expert Opin. Biol. Ther. 14 (2), 247–259. 10.1517/14712598.2014.857653 24397854

[B120] Schulz C. Perdiguero E. G. Chorro L. Szabo-Rogers H. Cagnard N. Kierdorf K. (2012). A lineage of myeloid cells independent of myb and hematopoietic stem cells. Science 336 (6077), 86–90. 10.1126/science.1219179 22442384

[B121] Seitz J. M. Eifler R. Bach F. W. Maier H. J. (2014). Magnesium degradation products: effects on tissue and human metabolism. J. Biomed. Mater Res. A 102 (10), 3744–3753. 10.1002/jbm.a.35023 24222399

[B122] Shapouri-Moghaddam A. Mohammadian S. Vazini H. Taghadosi M. Esmaeili S. Mardani F. (2018). Macrophage plasticity, polarization, and function in health and disease. J. Cell Physiol. 233 (9), 6425–6440. 10.1002/jcp.26429 PMC13482560 29319160

[B123] Shen H. Xu B. Yang C. Xue W. You Z. Wu X. (2022). A DAMP-scavenging, IL-10-releasing hydrogel promotes neural regeneration and motor function recovery after spinal cord injury. Biomaterials 280, 121279. 10.1016/j.biomaterials.2021.121279 34847433

[B124] Solier S. Müller S. Cañeque T. Versini A. Mansart A. Sindikubwabo F. (2023). A druggable copper-signalling pathway that drives inflammation. Nature 617 (7960), 386–394. 10.1038/s41586-023-06017-4 37100912 PMC10131557

[B125] Spiller K. L. Nassiri S. Witherel C. E. Anfang R. R. Ng J. Nakazawa K. R. (2015). Sequential delivery of immunomodulatory cytokines to facilitate the M1-to-M2 transition of macrophages and enhance vascularization of bone scaffolds. Biomaterials 37, 194–207. 10.1016/j.biomaterials.2014.10.017 25453950 PMC4312192

[B126] Sridharan R. Cavanagh B. Cameron A. R. Kelly D. J. O’Brien F. J. (2019). Material stiffness influences the polarization state, function and migration mode of macrophages. Acta Biomater. 89, 47–59. 10.1016/j.actbio.2019.02.048 30826478

[B127] Stöger J. L. Gijbels M. J. J. Velden S. van der Manca M. van der Loos C. M. Biessen E. A. (2012). Distribution of macrophage polarization markers in human atherosclerosis. Atherosclerosis 225 (2), 461–468. 10.1016/j.atherosclerosis.2012.09.013 23078881

[B128] Sun Y. Liu T. Hu H. Xiong Z. Zhang K. He X. (2022). Differential effect of tantalum nanoparticles *versus* tantalum micron particles on immune regulation. Mater Today Bio 16, 100340. 10.1016/j.mtbio.2022.100340 PMC9278074 35847379

[B129] Sunderkötter C. Goebeler M. Schulze-Osthoff K. Bhardwaj R. Sorg C. (1991). Macrophage-derived angiogenesis factors. Pharmacol. Ther. 51 (2), 195–216. 10.1016/0163-7258(91)90077-y 1784630

[B130] Tamura R. Tanaka T. Yamamoto Y. Akasaki Y. Sasaki H. (2018). Dual role of macrophage in tumor immunity. Immunotherapy 10 (10), 899–909. 10.2217/imt-2018-0006 30073897

[B131] Tang Y. Wu C. Wu Z. Hu L. Zhang W. Zhao K. (2017). Fabrication and *in vitro* biological properties of piezoelectric bioceramics for bone regeneration. Sci. Rep. 7, 43360. 10.1038/srep43360 28240268 PMC5327417

[B132] Tao D. Wang H. Chang S. Cheng J. Da N. Zhang L. (2025). Matrix viscoelasticity orchestrates osteogenesis *via* mechanotransduction mediated metabolic switch in macrophages. Adv. Healthc. Mater 14 (11), e2405097. 10.1002/adhm.202405097 40042258 PMC12023826

[B133] Toledano M. Vallecillo-Rivas M. Osorio M. T. Muñoz-Soto E. Toledano-Osorio M. Vallecillo C. (2021). Zn-Containing membranes for guided bone regeneration in dentistry. Polym. (Basel) 13 (11), 1797. 10.3390/polym13111797 PMC8199215 34072433

[B134] Trinchieri G. Gerosa F. (1996). Immunoregulation by interleukin-12. J. Leukoc. Biol. 59 (4), 505–511. 10.1002/jlb.59.4.505 8613697

[B135] Tu P. cheng Pan Y. lan Liang Z. qing Yang G. l. Wu C. j. Zeng L. (2022). Mechanical stretch promotes macrophage polarization and inflammation *via* the RhoA-ROCK/NF-κB pathway. BioMed Res. Int. 2022, 6871269. 10.1155/2022/6871269 35915804 PMC9338847

[B136] Vassey M. Ma L. Kämmerling L. Mbadugha C. Trindade G. F. Figueredo G. P. (2023). Innate immune cell instruction using micron-scale 3D objects of varied architecture and polymer chemistry: the ChemoArchiChip. Matter 6 (3), 887–906. 10.1016/j.matt.2023.01.002

[B137] Vi L. Baht G. S. Whetstone H. Ng A. Wei Q. Poon R. (2015). Macrophages promote osteoblastic differentiation *in vivo:* implications in fracture repair and bone homeostasis. J. Bone Mineral Res. 30 (6), 1090–1102. 10.1002/jbmr.2422 25487241

[B138] Wan Z. Shin L. Y. Wang Y. F. Huang Z. Dong Y. Lee C. W. (2020). Role of skeletal macrophages in fracture repair: a systematic review. Biomed. Rep. 13 (6), 1. 10.3892/br.2020.1360 33082950 PMC7560542

[B139] Wang C. Ma C. Gong L. Guo Y. Fu K. Zhang Y. (2021a). Macrophage polarization and its role in liver disease. Front. Immunol. 12, 803037. 10.3389/fimmu.2021.803037 34970275 PMC8712501

[B140] Wang C. Xu D. Lin L. Li S. Hou W. He Y. (2021b). Large-pore-size Ti6Al4V scaffolds with different pore structures for vascularized bone regeneration. Mater Sci. Eng. C Mater Biol. Appl. 131, 112499. 10.1016/j.msec.2021.112499 34857285

[B141] Wang L. X. Zhang S. X. Wu H. J. Rong X. L. Guo J. (2019). M2b macrophage polarization and its roles in diseases. J. Leukoc. Biol. 106 (2), 345–358. 10.1002/JLB.3RU1018-378RR 30576000 PMC7379745

[B142] Wang X. Liu W. Yu X. Wang B. Xu Y. Yan X. (2022). Advances in surface modification of tantalum and porous tantalum for rapid osseointegration: a thematic review. Front. Bioeng. Biotechnol. 10, 983695. 10.3389/fbioe.2022.983695 36177183 PMC9513364

[B143] Wang Y. Pan Z. Wang Q. Shu Y. Tan Z. Chen Y. (2025). Sequential SDF-1/CGRP-releasing smart composite hydrogel promotes osteoporotic fracture healing by targeting sensory nerve-regulated bone remodeling. Mater Today Bio 32. 10.1016/j.mtbio.2025.101750 PMC12054128 40331153

[B144] Wermuth P. J. Jimenez S. A. (2015). The significance of macrophage polarization subtypes for animal models of tissue fibrosis and human fibrotic diseases. Clin. Transl. Med. 4, 2. 10.1186/s40169-015-0047-4 25852818 PMC4384891

[B145] Wissing T. B. Bonito V. van Haaften E. E. van Doeselaar M. Brugmans M. M. C. P. Janssen H. M. (2019). Macrophage-driven biomaterial degradation depends on scaffold microarchitecture. Front. Bioeng. Biotechnol. 7, 87. 10.3389/fbioe.2019.00087 31080796 PMC6497794

[B146] Woodhall B. Spurling R. G. (1945). Tantalum cranioplasty for war wounds of the skull. Ann. Surg. 121 (5), 649–671. 10.1097/00000658-194505000-00009 17858600 PMC1618271

[B147] Wosik J. Chen W. Qin K. Ghobrial R. M. Kubiak J. Z. Kloc M. (2018). Magnetic field changes macrophage phenotype. Biophysical J. 114 (8), 2001–2013. 10.1016/j.bpj.2018.03.002 PMC5937143 29694876

[B148] Wu C. L. McNeill J. Goon K. Little D. Kimmerling K. Huebner J. (2017). Conditional macrophage depletion increases inflammation and does not inhibit the development of osteoarthritis in Obese macrophage fas-induced apoptosis-transgenic mice. Arthritis Rheumatol. 69 (9), 1772–1783. 10.1002/art.40161 28544542 PMC5611814

[B149] Wu H. Dong H. Tang Z. Chen Y. Liu Y. Wang M. (2023). Electrical stimulation of piezoelectric BaTiO3 coated Ti6Al4V scaffolds promotes anti-inflammatory polarization of macrophages and bone repair *via* MAPK/JNK inhibition and OXPHOS activation. Biomaterials 293. 10.1016/j.biomaterials.2022.121990 36586147

[B150] Wynn T. A. Chawla A. Pollard J. W. (2013). Macrophage biology in development, homeostasis and disease. Nature 496 (7446), 445–455. 10.1038/nature12034 23619691 PMC3725458

[B151] Wynn T. A. Vannella K. M. (2016). Macrophages in tissue repair, regeneration, and fibrosis. Immunity 44 (3), 450–462. 10.1016/j.immuni.2016.02.015 26982353 PMC4794754

[B152] Xu A. T. Xie Y. W. Xu J. G. Li J. Wang H. He F. M. (2021). Effects of strontium-incorporated micro/nano rough titanium surfaces on osseointegration *via* modulating polarization of macrophages. Colloids Surf. B Biointerfaces 207, 111992. 10.1016/j.colsurfb.2021.111992 34391168

[B153] Xu J. Jia Y. Huang W. Shi Q. Sun X. Zheng L. (2022). Non-contact electrical stimulation as an effective means to promote wound healing. Bioelectrochemistry 146, 108108. 10.1016/j.bioelechem.2022.108108 35366594

[B154] Xu Z. Wu L. Tang Y. Xi K. Tang J. Xu Y. (2023). Spatiotemporal regulation of the bone immune microenvironment *via* dam-like biphasic bionic periosteum for bone regeneration. Adv. Healthc. Mater 12 (1). 10.1002/adhm.202201661 PMC11469314 36189833

[B155] Xuan Y. Li L. Ma M. Cao J. Zhang Z. (2022). Hierarchical intrafibrillarly mineralized collagen membrane promotes guided bone regeneration and regulates M2 macrophage polarization. Front. Bioeng. Biotechnol. 9. 10.3389/fbioe.2021.781268 PMC8826568 35155400

[B156] Yang P. Peng Y. Dai X. Jie J. Kong D. Gu X. (2023a). Bionic peptide scaffold *in situ* polarization and recruitment of M2 macrophages to promote peripheral nerve regeneration. Bioact. Mater. 30, 85–97. 10.1016/j.bioactmat.2023.07.003 37575879 PMC10412994

[B157] Yang X. Gao J. Yang S. Wu Y. Liu H. Su D. (2023b). Pore size-mediated macrophage M1 to M2 transition affects osseointegration of 3D-printed PEEK scaffolds. Int. J. Bioprint 9 (5), 755. 10.18063/ijb.755 37457949 PMC10339443

[B158] Yang Y. Guo Z. Chen W. Wang X. Cao M. Han X. (2021). M2 macrophage-derived exosomes promote angiogenesis and growth of pancreatic ductal adenocarcinoma by targeting E2F2. Mol. Ther. 29 (3), 1226–1238. 10.1016/j.ymthe.2020.11.024 33221435 PMC7934635

[B159] You J. Zhang Y. Zhou Y. (2022). Strontium functionalized in biomaterials for bone tissue engineering: a prominent role in osteoimmunomodulation. Front. Bioeng. Biotechnol. 10. 10.3389/fbioe.2022.928799 PMC9298737 35875505

[B160] Yuan P. Luo Y. Luo Y. Ma L. (2021). A “sandwich” cell culture platform with NIR-Responsive dynamic stiffness to modulate macrophage phenotypes. Biomater. Sci. 9 (7), 2553–2561. 10.1039/D0BM02194F 33576368

[B161] Yue Y. Yang X. Feng K. Wang L. Hou J. Mei B. (2017). M2b macrophages reduce early reperfusion injury after myocardial ischemia in mice: a predominant role of inhibiting apoptosis *via* A20. Int. J. Cardiol. 245, 228–235. 10.1016/j.ijcard.2017.07.085 28764858

[B162] Yunna C. Mengru H. Lei W. Weidong C. (2020). Macrophage M1/M2 polarization. Eur. J. Pharmacol. 877, 173090. 10.1016/j.ejphar.2020.173090 32234529

[B163] Zamani D. Moztarzadeh F. Bizari D. (2019). Alginate-bioactive glass containing Zn and Mg composite scaffolds for bone tissue engineering. Int. J. Biol. Macromol. 137, 1256–1267. 10.1016/j.ijbiomac.2019.06.182 31279876

[B164] Zelová H. Hošek J. (2013). TNF-α signalling and inflammation: interactions between old acquaintances. Inflamm. Res. 62 (7), 641–651. 10.1007/s00011-013-0633-0 23685857

[B165] Zengo A. N. Pawluk R. J. Bassett C. A. (1973). Stress-induced bioelectric potentials in the dentoalveolar complex. Am. J. Orthod. 64 (1), 17–27. 10.1016/0002-9416(73)90277-7 4515022

[B166] Zhang H. Wang R. Wang G. Zhang B. Wang C. Li D. (2021). Single-cell RNA sequencing reveals B cells are important regulators in fracture healing. Front. Endocrinol. (Lausanne) 12, 666140. 10.3389/fendo.2021.666140 34819916 PMC8606664

[B167] Zhang X. Wang T. Zhang Z. Liu H. Li L. Wang A. (2023). Electrical stimulation system based on electroactive biomaterials for bone tissue engineering. Mater. Today 68, 177–203. 10.1016/j.mattod.2023.06.011

[B168] Zhao Z. Hou X. Yin X. Li Y. Duan R. Boyce B. F. (2015). TNF induction of NF-κB RelB enhances RANKL-induced osteoclastogenesis by promoting inflammatory macrophage differentiation but also limits it through suppression of NFATc1 expression. PLOS ONE 10 (8), e0135728. 10.1371/journal.pone.0135728 26287732 PMC4545392

[B169] Zheng X. Chen L. Tan J. Miao J. Liu X. Yang T. (2022). Effect of micro/nano-sheet array structures on the osteo-immunomodulation of macrophages. Regen. Biomater. 9, rbac075. 10.1093/rb/rbac075 36284748 PMC9580515

[B170] Zhou Y. W. Wu Y. (2022). Substrate viscoelasticity amplifies distinctions between transient and persistent LPS-induced signals. Adv. Healthc. Mater 11 (8), e2102271. 10.1002/adhm.202102271 34855279

[B171] Zhu S. Zhou J. Xie Z. (2024). The balance between helper T 17 and regulatory T cells in osteoimmunology and relevant research progress on bone tissue engineering. Immun. Inflamm. Dis. 12 (9), e70011. 10.1002/iid3.70011 39264247 PMC11391570

[B172] Zhu X. Zhao Y. Liu Y. Shi W. Yang J. Liu Z. (2023). Macrophages release IL11-containing filopodial tip vesicles and contribute to renal interstitial inflammation. Cell Commun. Signal. 21 (1), 293. 10.1186/s12964-023-01327-6 37853428 PMC10585809

[B173] Zhuang Z. Zhang Y. Sun S. Li Q. Chen K. An C. (2020). Control of matrix stiffness using methacrylate-gelatin hydrogels for a macrophage-mediated inflammatory response. ACS Biomater. Sci. Eng. 6 (5), 3091–3102. 10.1021/acsbiomaterials.0c00295 33463297

